# Mitochondrial Lon regulates apoptosis through the association with Hsp60–mtHsp70 complex

**DOI:** 10.1038/cddis.2015.9

**Published:** 2015-02-12

**Authors:** T-Y Kao, Y-C Chiu, W-C Fang, C-W Cheng, C-Y Kuo, H-F Juan, S-H Wu, A Y-L Lee

**Affiliations:** 1 Department of Medical Laboratory Science and Biotechnology, Yuanpei University, Hsinchu 300, Taiwan; 2 National Institute of Cancer Research, National Health Research Institutes, Zhunan, Miaoli 35053, Taiwan; 3 Institute of Biological Chemistry, Academia Sinica, Taipei 115, Taiwan; 4 Institute of Biochemical Sciences, National Taiwan University, Taipei 106, Taiwan; 5 Institute of Molecular and Cellular Biology, National Taiwan University, Taipei 106, Taiwan; 6 Department of Life Science, National Taiwan University, Taipei 106, Taiwan; 7 Department of Chemistry, National Taiwan University, Taipei 106, Taiwan; 8 Graduate Institute of Basic Medical Science, China Medical University, Taichung 404, Taiwan

## Abstract

Human Lon protease is a mitochondrial matrix protein with several functions, including protein degradation, mitochondrial DNA (mtDNA) binding, and chaperone activity. Lon is currently emerging as an important regulator of mitochondria-contributed tumorigenesis due to its overexpression in cancer cells. To understand the mechanism of increased Lon in tumor cells, we studied the interactome to identify the chaperone Lon-associated proteins by proteomics approaches using the cells overexpressing Lon. In the present study, we designed a method connecting co-immunoprecipitation (Co-IP) to in-solution digestion for the shotgun mass spectrometry. We identified 76 proteins that were putative Lon-associated proteins that participated in mitochondrial chaperone system, cellular metabolism and energy, cell death and survival, and mtDNA stability. The association between Lon and NDUFS8 or Hsp60–mtHsp70 complex was confirmed by Co-IP and immunofluorescence co-localization assay. We then found that the protein stability/level of Hsp60–mtHsp70 complex depends on the level of Lon under oxidative stress. Most importantly, the ability of increased Lon-inhibited apoptosis is dependent on Hsp60 that binds p53 to inhibit apoptosis. These results suggest that the mechanism underlying cell survival regulated by Lon is mediated by the maintenance of the protein stability of Hsp60–mtHsp70 complex. This new knowledge of chaperone Lon interactome will allow us to better understand the cellular mechanism of Lon in mitochondrial function and of its overexpression in enhancing cell survival and tumorigenesis.

Under stress circumstances, proteins are at risk of being inactivated by misfolding, unfolding, or aggregation. Protein quality control (PQC) system, chaperones and proteases, safeguards the function under cellular stress conditions. The coordinated function of the two components is required to stabilize misfolded proteins and refold or remove them to avoid the deleterious effects of protein aggregation.^1, 2^

Lon is a highly conserved AAA+ (ATPases associated with a variety of cellular activities) protease and is committed to several crucial functions, including adenosine-5′-triphosphate (ATP)-dependent proteolytic, DNA binding, and chaperone-like activity.^3, 4, 5^ Eukaryotic Lon protease operates in PQC in mitochondria by its multiple functions^4, 6, 7^ and has a critical role in the maintenance of mitochondrial function, biogenesis, and homeostasis.^8^ Mitochondria orchestrate the process of cell life and death, thereby employing a decisive control over signaling leading to cellular survival, in particular the intrinsic pathway of apoptosis.^9^ Thus it is not surprising that the level of Lon regulates mitochondrial functions that contribute to cell fate and survival. Indeed, Lon downregulation leads to loss of mitochondrial function, early embryonic lethality, reduced cell proliferation, and apoptosis.^10, 11, 12, 13^ Lon upregulation is critical for cancer cell survival and tumorigenesis by regulating stress responses induced by oxidative condition.^11, 12^ Lon is a stress protein and induced by a number of stresses such as hypoxia and oxidative and mitochondrial unfolded protein stress,^11, 14, 15, 16, 17^ which are common stress phenotypes of cancer cells. During hypoxia, Lon is upregulated by the hypoxia-inducible factor-1*α* and is involved in a mechanism to respond to low oxygen availability and adapt cancer cells to a hypoxic environment.^16^ In addition to its proteolytic activity, Lon has been found to show chaperone properties.^3, 11, 15^ Lon promotes the assembly of cytochrome *c* oxidase (COX) 4–1 subunits, suggesting that Lon has chaperone activity in yeast and mammalian cells.^15, 18^ Molecular chaperones of heat-shock protein (HSP) family have important roles in promoting tumor growth and survival.^19, 20^ Thus mitochondrial Lon may be a protein chaperone to assist cells to survive and adapt to various stresses that are linked to oncogenesis. However, very few human Lon chaperone clients have been identified, and the mechanism of how upregulated Lon employs its chaperone activity to regulate apoptosis remains obscure.

To study the roles of Lon overexpression in cancer cell survival, we utilized proteomic techniques to identify chaperone Lon-interacting proteins. The interactome suggests that Lon may participate in many cellular activities, including mitochondrial chaperones, cellular metabolism and energy, Redox regulation, cell death and survival, and mitochondrial DNA (mtDNA) stability. We identified heat-shock protein 60 (Hsp60), mtHsp70, and NDUFS8 (NADH dehydrogenase [ubiquinone] iron-sulfur protein 8) as Lon-interacting proteins by using co-immunoprecipitation (Co-IP) and immunofluorescence experiments. We further characterized that the protein stability of Hsp60 and mtHsp70 depends on the level of Lon under oxidative stress. We know the fact that Hsp60 and mtHsp70 forms a complex^21, 22^ and are overexpressed in cancer cells and have crucial roles in modulating the apoptotic pathways and in cancer development.^19^ Consistently, Hsp60 is essential to maintain apoptosis inhibition preserved by Lon overexpression. These results suggest that the mechanism underlying apoptosis regulated by Lon is mediated by the maintenance of the stability of Hsp60–mtHsp70 complex.

## Results

### Lon protein level regulates cell survival and apoptosis under environmental stresses

**Figure 1:**
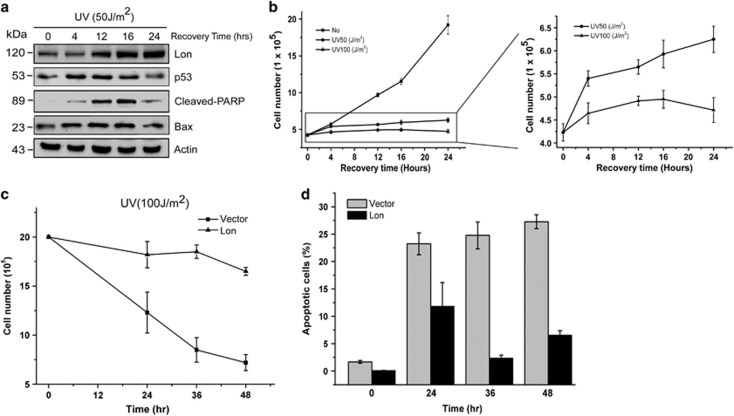
The level of Lon is associated with cell survival under UV irradiation. (**a**) 293 cells were treated with UV (50 J/m^2^) and collected after the indicated recovery time. The apoptosis induction was quantified by the activation of p53 and Bax as well as cleavage of PARP. The expression was detected by western blotting using the indicated antibodies. An antibody to actin was used as a loading control. (**b**) The viable cells were determined by the trypan blue dye exclusion assay. (**c**) The overexpression of Lon promotes cell survival under UV irradiation. 293 cells overexpressing Lon or not were treated with UV (100 J/m^2^) and collected after the indicated recovery time. The viable cells were determined by the trypan blue dye exclusion assay. (**d**) The overexpression of Lon inhibits apoptosis under UV treatment. After 24-h transfection with Lon-expressing plasmid or not, 293 cells were treated with UV (100 J/m^2^) and collected after the indicated recovery time. Apoptotic cells were detected by Annexin V/PI staining using flow cytometry. Data are presented as mean±S.D. of at least three independent experiments

We previously showed that Lon expression is greatly induced by hydrogen peroxide (H_2_O_2_), hypoxia, and ultra-violet (UV) irradiation and that increased levels of Lon inhibits apoptosis and enhances cell survival under these stresses.^11^ Indeed, Lon protein was upregulated starting from 12 to 24 h after UV treatment (50 J/m^2^), and opposite kinetics was observed in pro-apoptotic proteins, p53, cleaved PARP, and Bax (Figure 1a), suggesting that Lon may act as a stress-induced protein to protect cells from apoptosis under UV stress. Similarly, the number of viable cells obviously increased in a recovery time-dependent manner under the treatment of 50 J/m^2^ UV (Figure 1b, right panel). To further confirm that Lon will protect cells from apoptosis under UV stress, we overexpressed Lon in cells, treated the cells with UV stress (100 J/m^2^), and then examined the cell viability. The survived cells were largely decreased in the vector cells in a time-dependent manner but not in the Lon-overexpressed cells (Figure 1c). Consistently, apoptotic cells detected by Annexin V/Propidium Iodide (PI) staining were increased in the vector cells but not in Lon-overexpressed cells (Figure 1d). These data confirmed that upregulation of Lon protein inhibits apoptosis and promotes cell survival under environmental stresses.

### Proteome-wide identification of client proteins of chaperone Lon

**Figure 2:**
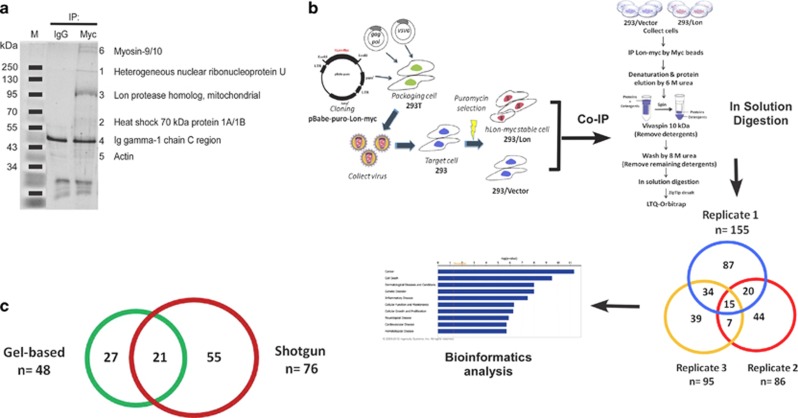
Identification of chaperone Lon-associated proteins by proteomic approach. (**a**) Identification of Lon-associated proteins by gel-based proteomics. IP eluate was analyzed on one-dimensional gel (1-D gel) using sypro ruby staining. Protein bands obtained from 1-D gel were identified by mass spectrometry. The proteins with the highest Mascot score are shown. (**b**) The flow chart of in-solution digestion shotgun proteomics. The stable 293 cells overexpressing Lon (293/Lon) were used to identify associated proteins of chaperone Lon. The immunocomplex of Lon-myc was pulled down by myc agarose beads, which can avoid the contamination of antibodies. Immunocomplex was then eluted by 6 M urea, and detergents were removed from the complex by vivaspin centrifugation and by 8 M urea wash. After Mass analysis, the Venn diagram of the coverage and overlap of the results from three independent experiments are shown. Functional analyses were generated through Ingenuity Pathways Analysis (Ingenuity Systems, http://www.ingenuity.com). (**c**) Overlap of the result between gel-based proteomics and in-solution digestion shotgun proteomics

To investigate the role of chaperone Lon in cell survival under environmental stresses, we used the stable 293 cells overexpressing Lon (293/Lon) to identify the associated proteins of chaperone Lon by proteomic approach, by which we could exclude most substrates of Lon protease. The expression and cellular localization of Lon-myc in 293/Lon were checked (Supplementary Figures S1A and B). An Co-IP approach was performed to purify chaperone Lon-associated proteins using anti-myc antibodies. The purified proteins were resolved by 1-D SDS-PAGE (Figure 2a). A total of 48 proteins were identified with high confidence, including mitochondrial Lon protease and mitochondrial heat-shock protein 70 (mtHsp70/mortalin) (Figure 2a and Supplementary Table S1). To confirm and identify more Lon-associated proteins, we developed a shotgun proteomic approach that combines Co-IP and in-solution digestion to replace the step of in-gel digestion. The flow chart of the shotgun approach is shown in Figure 2b. To remove the contamination of antibodies in the Co-IP experiment, the IP-purified proteins by agarose-conjugated anti-myc beads were eluted by 6 M urea, a strong protein denaturant. However, anti-myc antibodies were not eluted by 6 M urea but by boiling at 95 °C, because they were covalently conjugated with agarose beads (Supplementary Figures S2A and B). Three independent experiments were performed, and each consisted of a negative control sample from 293/myc cells (Supplementary Tables S2–S4). Totally, 246 proteins were identified, (Figure 2b) and 76 proteins were found at least twice in three independent experiments (Supplementary Table S5). Proteins detectable in any one of these three independent negative control samples were eliminated from our list (Supplementary Figure S3A). Furthermore, 21 Lon-associated proteins were found on both lists of gel-based and shotgun proteomic analysis, including mitochondrial Lon protease, mtHsp70, Myosin-9/10, 78 kDa glucose-regulated protein (GRP78),^23^ and protocadherin-18 (Figure 2c and Supplementary Table S6).

### Characterization and validation of potential associated proteins of mitochondrial chaperone Lon

To validate the results from the proteomic study, we first performed bioinformatic analyses of the function of 246 Lon-associated proteins. Ingenuity Pathway Analysis (IPA, Ingenuity Systems, http://www.ingenuity.com) showed that many of them are associated with cancer and cell death (Supplementary Figure S3B). Table 1 shows the identified proteins from mitochondria, which were grouped according to their cellular location and biological functions by literature investigation. They are classified in various biological function groups, including mitochondrial chaperones, cellular metabolism and energy, Redox regulation, cell death and survival, and mtDNA stability. From Table 1, we found that NDUFS8, located in the inner membrane of mitochondria, was on the candidate list. NDUFS8 was shown to be a Lon-regulated protein in our previous study, which interacts with Lon and its protein stability or level is regulated by Lon.^11^ The interaction between Lon and NDUFS8 was confirmed by Co-IP experiments (Supplementary Figure S4A), and confocal immunofluorescence analysis (Supplementary Figure S4B), establishing a reliability of the candidate list.

### Mitochondrial Lon interacts with Hsp60–mtHsp70 complex

**Figure 3:**
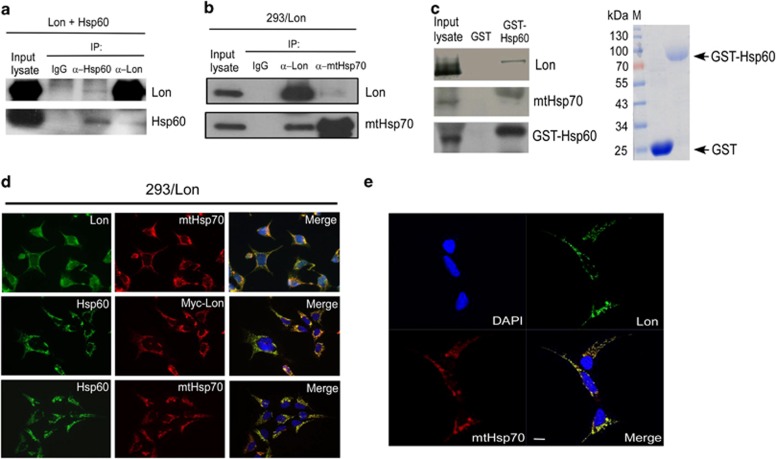
Lon interacts with Hsp60–mtHsp70 complex. (**a**) 293 cells were transiently transfected with plasmid encoding Hsp60 and Lon, and cell extracts were co-immunoprecipitated with anti-Hsp60 and anti-Lon, respectively, and analyzed by western blotting using the indicated antibodies. (**b**) 293/Lon cells were directly co-immunoprecipitated by anti-mtHsp70 or anti-Lon to further validate the interaction between endogenous mtHsp70 and Lon. Western blotting was performed by using the indicated antibodies. (**c**) Direct interaction between Hsp60 and Lon was verified by glutathione *S*-transferase (GST) pull-down assay. Approximately 0.3 *μ*g of the GST proteins were added and shown on sodium dodecyl sulfate-polyacrylamide gel electrophoresis stained with Coomassie brilliant blue (right panel). (**d**) Immunofluorescence assay was applied to verify the protein–protein interaction between Lon and Hsp60–mtHsp70 complex. 293/Lon cells were immunostained by anti-Lon (green) and anti-mtHsp70 (red) or anti-Hsp60 (green) and anti-myc (red) antibodies, respectively. (**e**) The interaction of Lon with mtHsp70 was validated by confocal immunofluorescence. 293/Lon cells were immunostained by anti-Lon (green) and anti-mtHsp70 (red) following image capturing by confocal microscopy. Scale bar, 10 *μ*m

Hsp60 and mtHsp70 as Lon-associated proteins that were found on both lists of gel-based and shotgun proteomic analysis (Supplementary Tables S5 and S6). In addition, protein network analysis by MetaCore (GeneGo, St Joseph, MI, USA) showed that mtHsp70 and Hsp60 were found in the same interaction network (Supplementary Figure S3C). Indeed, mtHsp70 was shown to directly bind with Hsp60, and Hsp60–mtHsp70 complex participates in the translocation and folding/assembly of nuclear-encoded proteins in the mitochondrial matrix.^7, 21, 22^ The Hsp60–mtHsp70 complex is also involved in cell death and survival as well as mtDNA stability.^24, 25, 26^ Therefore we then tested whether Lon is a component of the Hsp60–mtHsp70 complex. The association between Lon and Hsp60–mtHsp70 complex was first examined by Co-IP experiment. The result showed that Lon co-immunoprecipitated with Hsp60 and *vice versa* (Figure 3a), and endogenous mtHsp70 was able to be co-immunoprecipitated with Lon (Figure 3b), suggesting that Lon is a component of Hsp60–mtHsp70 complex *in vivo*. Glutathione *S*-transferase pull-down assay confirmed a direct interaction between HSP60 and Lon *in vitro* (Figure 3c). Immunofluorescence assay showed that Lon was colocalized with mtHsp70 and Hsp60 in either 293/Lon cells (Figure 3d) or 293 cells under oxidative stress (Supplementary Figure S5), in which the interaction between Hsp60 and mtHsp70 was used as a positive control. Finally, a confocal immunofluorescence analysis confirmed the colocalization and excluded the possibility that the merged signals come from overlapping of different plane signals in three-dimensional space (Figure 3e). These data verified the association between mitochondrial Lon and Hsp60–mtHsp70 complex in cells.

### Mitochondrial Lon contributes to the stability of Hsp60–mtHsp70 complex

**Figure 4:**
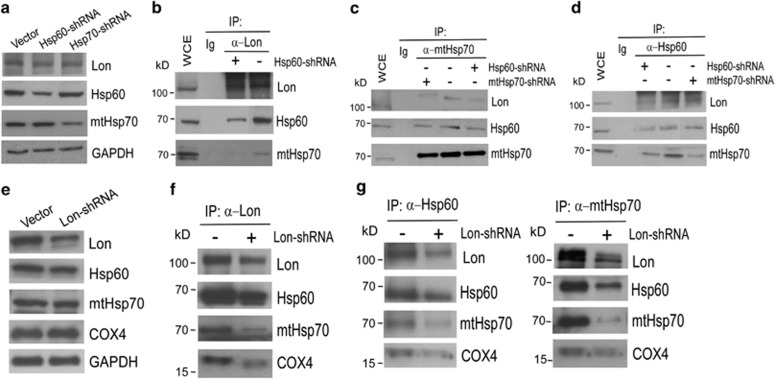
Characterization of the interaction between Lon and Hsp60–mtHsp70 complex. FADU cells were lentivirally infected with vector or vector expressing shRNAs for Hsp60, mtHsp70, or Lon. (**a** and **e**) The expression of Hsp60, mtHsp70, and Lon is shown. The cells were directly co-immunoprecipitated by (**b** and **f**) anti-Lon, (**c** and **g**) anti-mtHsp70, or (**d** and **g**) anti-Hsp60 to examine the interaction between endogenous Lon and either Hsp60 or mtHsp70 as well as the interaction between Hsp60 and mtHsp70. Western blotting was performed by using the indicated antibodies

To characterize the interaction between Lon and Hsp60–mtHsp70 complex, we first knocked down the expression of Hsp60 and mtHsp70 by shRNA to examine the effect on their interaction. The downregulation of Hsp60 and mtHsp70 were not able to affect the protein level of Lon (Figure 4a). We found that the downregulation of Hsp60 decreases the binding ability of Lon with mtHsp70 according to the Co-IP experiment (Figures 4b and c). However, the downregulation of mtHsp70 has no significant effect on the binding ability of Lon with Hsp60 (Figure 4d). We then knocked down the expression of Lon by shRNA to examine the effect on the interaction. The downregulation of Lon was not able significantly to affect the protein level of Hsp60 and mtHsp70 either (Figure 4e). Consistently, the downregulation of Lon decreases the binding ability of Lon with Hsp60 and mtHsp70 (Figure 4f), confirming the specificity of the interaction of Lon with Hsp60–mtHsp70 complex. In addition, the interaction in mitochondrial distribution was confirmed by the detection of COX4 (Figure 4f), which is located in the complex IV of respiratory chain and is a binding client of mtHsp70.^27^ We found that the downregulation of Lon decreases the binding ability of Hsp60 with mtHsp70 (Figure 4g). These findings suggest that mitochondrial Lon has an essential role in the stability of Hsp60–mtHsp70 complex and potentially suggest a critical role of Lon–Hsp60 interaction in the molecular function of Lon–Hsp60–mtHsp70 complex in mitochondria.

### Functional characterization of association between Lon and Hsp60–mtHsp70 complex

**Figure 5:**
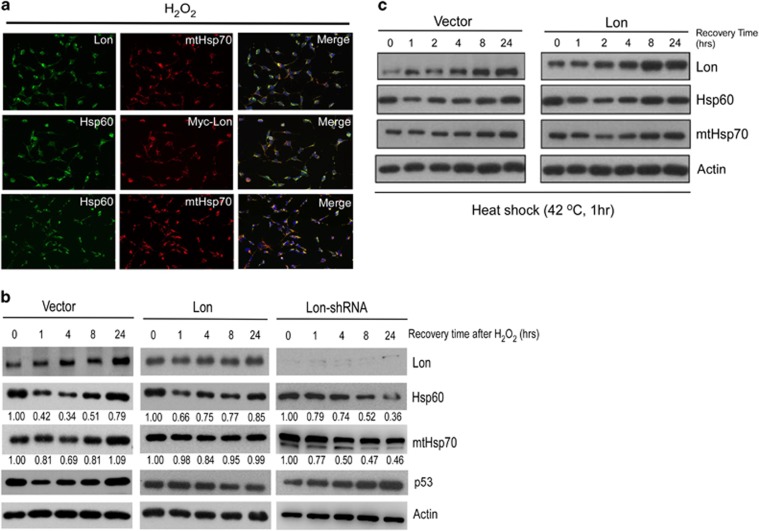
Characterization of the interaction between Lon and Hsp60–mtHsp70 complex under stress. (**a**) Interaction between Lon and Hsp60–mtHsp70 under oxidative stress. 293/Lon cells were exposed to 200 *μ*M H_2_O_2_ for 1 h and directly stained by anti-Lon (green), anti-mtHsp70 (red), anti-Hsp60 (green), or anti-myc (red) as indicated, following image capture by fluorescence microscopy. DNA was stained with DAPI (4,6-diamidino-2-phenylindole; blue). (**b**) The protein level of Hsp60 and mtHsp70 is dependent on the level of Lon under oxidative stress. Indicated 293 cells were treated with 200 *μ*M H_2_O_2_ for 1 h and then left to recover for 1, 4, 8, and 24 h after treatment. Western blotting was performed by using the indicated antibodies, and anti-actin antibody was used as a loading control. The number represents the band intensity normalized against actin. (**c**) Lon enhances the stability of Hsp60 and mtHsp70 under heat-shock stress. Indicated 293 cells are treated with 42 °C for 1 h and then left to recover for 1, 4, 8, and 24 h after treatment. Immunoblots were probed with the indicated antibodies, and anti-actin antibody was used as a loading control

Lon and Hsp60–mtHsp70 complex are stress proteins, and they are responsible for the PQC in mitochondria under cellular stress, such as UV and oxidative stress.^7, 11^ Thus we tested whether Lon interacts with Hsp60–mtHsp70 complex under oxidative stress. Immunofluorescence analysis showed that Lon is associated with Hsp60–mtHsp70 complex under oxidative stress (Figure 5a), suggesting that Lon and Hsp60–mtHsp70 complex function together under oxidative condition. To investigate the functional roles of their association, we first examined whether Lon regulates the stability or level of Hsp60–mtHsp70 complex after oxidative stress challenge. In wild-type 293/Vector cells, Lon level was considerably induced starting from 4 h after H_2_O_2_ treatment, which is consistent with the previous findings.^11, 14^ The expression pattern of Hsp60 and mtHsp70 was similar to that of Lon, which was increased after 4 h in a time-dependent manner (Figure 5b, left panel). In 293/Lon cells, the expression pattern of Hsp60 and mtHsp70 was similar to the wild-type cells but its kinetics was faster, which increased beginning from 1 h (Figure 5b, middle panel). We then knocked down the Lon expression by shRNA to perform the same experiments. The expression of Lon was suppressed by Lon-shRNA (sh-1) (Figure 5b, right panel; Supplementary Figure S6). In Lon-compromised cells, Hsp60 and mtHsp70 levels were downregulated beginning from 1 h after the treatment (Figure 5b, right panel). Lon upregulation also maintained the protein level of Hsp60 and mtHsp70 under heat shock (Figure 5c). These data suggest that the expression of Lon positively regulates the protein stability/level of Hsp60–mtHsp70 complex under environmental stress.

### Mitochondrial Lon regulates apoptosis via the interaction with Hsp60

**Figure 6:**
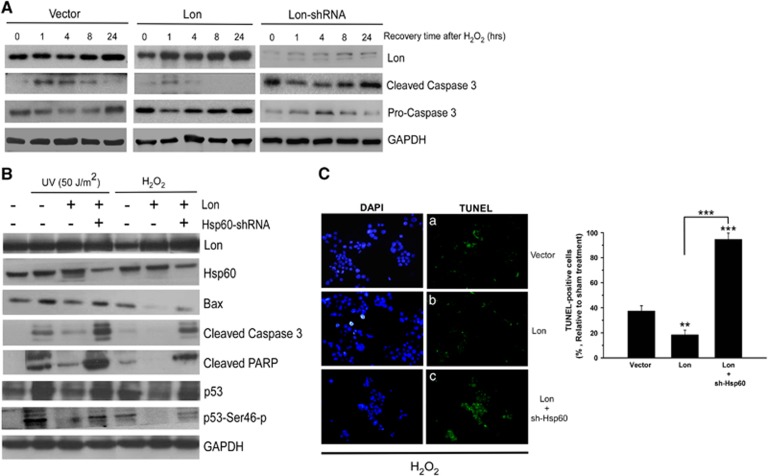
Mitochondrial Lon regulates apoptosis via the interaction with Hsp60–mtHsp70 complex under oxidative stress. (**A**) Indicated 293 cells were treated with 200 *μ*M H_2_O_2_ for 1 h and then left to recover for 1, 4, 8, and 24 h after treatment. Western blotting was performed by using the indicated antibodies, and anti-GAPDH (anti-glyceraldehyde 3-phosphate dehydrogenase) antibody was used as a loading control. (**B**) FADU and FADU-shHSP60 cells with or without Lon overexpression were treated with 200 *μ*M H_2_O_2_ for 4 h or UV (50 J/m^2^) and then recovered for 4 h. Apoptosis-associated proteins were detected by western blotting analysis, and anti-GAPDH antibody was used as a loading control. (**C**) FADU cells were transfected with pcDNA3-Lon and with or without lentiviral vector expressing shRNA-Hsp60, and the cells were treated with 200 *μ*M H_2_O_2_ for 4 h. TUNEL assay was applied to examine the effect of Hsp60 depletion on Lon-inhibited apoptosis. TUNEL-positive cells (green fluorescence) were counted under H_2_O_2_ treatment in the cells expressing vector (a), Lon (b) or Lon plus shRNA-Hsp60 (c). DAPI (4,6-diamidino-2-phenylindole) was used for nuclear staining. The error bars shown in the right panel represent the S.D. from three different experiments. **P<0.01 and ***P<0.001

We next investigated how Lon increases cell survival under stress through regulating apoptosis by binding with Hsp60–mtHsp70 complex. We found that Lon increases the protein stability or level of Hsp60 and mtHsp70 after cellular stress challenge in 293 cells, especially Hsp60 (Figures 5b and c). Under the same condition, the level of Lon expression was correlated to a decrease or increase in cleaved caspase 3 after H_2_O_2_ treatment (Figure 6a), suggesting that upregulation of Lon protects cells from apoptosis and downregulation of Lon induces apoptosis during recovery from oxidative stress. Combined together, the trend in Hsp60 and mtHsp70 level was reversely correlated to the one in cleaved caspase 3 and p53 apoptotic markers (Figures 5b and 6A), confirming that Hsp60 and mtHsp70 are involved in the regulation of apoptosis.^28, 29^ These results may suggest that upregulation of Lon enhances the protein stability/level of Hsp60–mtHsp70 and further protects cell from apoptosis under environmental stress through binding with Hsp60 or mtHsp70. To prove this, we knocked down the expression of Hsp60 by shRNA to examine the effect on apoptosis activation under Lon overexpression. The signals of pro-apoptotic proteins, Bax, cleaved caspase 3, cleaved PARP, p53, and phosphorylated p53^Ser46^, were activated after UV or H_2_O_2_ treatment in FADU cells (Figure 6B), suggesting that the treated cells were indeed undergoing apoptotic process. The levels of pro-apoptotic proteins were decreased and apoptosis was almost inhibited in the cells overexpressing Lon after stresses (Figure 6B), suggesting that Lon protein indeed is important for the regulation of apoptosis under environmental stresses. However, when we knocked down the expression of Hsp60, the levels of apoptotic proteins were increased back in the cells overexpressing Lon under stresses (Figure 6B). These results indicated that Hsp60 has an important role in the apoptosis regulation mediated by Lon under environmental stress. To confirm the role of Hsp60 in Lon-mediated apoptosis regulation, terminal deoxynucleotidyl transferase-mediated dUTP nick-end labeling (TUNEL) assay was performed under the same condition. The TUNEL-positive cells were detected less in Lon-overexpressed cells, compared with the vector control cells (a and b in Figure 6C). Similarly, the TUNEL-positive cells were largely increased in the cells overexpressing Lon when Hsp60 was knocked down (a and c in Figure 6C). These data indicated that increased Lon protein protects cell from apoptosis under environmental stress through interacting with and stabilizing Hsp60.

## Discussion

**Figure 7:**
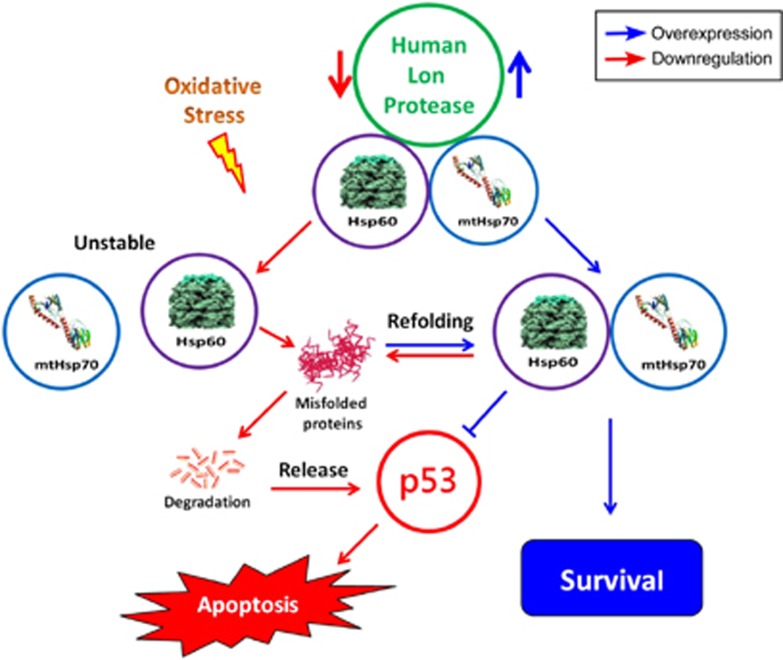
Human mitochondrial Lon interacts with Hsp60–mtHsp70 complex as a cytoprotective chaperone network to enhance cell survival under oxidative stress. Overexpression of Lon enhances the protein stability of Hsp60–mtHsp70 complex and inhibits apoptosis via stabilizing the Hsp60–p53 complex. Loss of Lon leads to a decrease in the interaction between Hsp60 and mtHsp70, suggesting that the protein stability of Hsp60–mtHsp70 complex is dependent on Lon. Hsp60–mtHsp70 complex has an essential role in Lon-mediated inhibition of apoptosis under oxidative stress. Through binding with Hsp60–mtHsp70 complex, increased mitochondrial Lon inhibits apoptosis and contributes to cell survival under oxidative stress

Mitochondrial Lon modulates the organelle function, cell proliferation, and apoptosis.^4, 10, 11, 12^ The upregulation of Lon is critical for cell survival under oxidative and hypoxic stress.^11, 14, 16^ In this study, via proteomic approach, we have shown that mitochondrial Lon interacts and functions together with Hsp60–mtHsp70 complex in apoptosis regulation. Loss of Lon leads to a decrease in protein levels of Hsp60 and mtHsp70 under oxidative stress, suggesting that the protein stability of Hsp60–mtHsp70 complex is dependent on Lon. In addition, loss of Hsp60 leads to the instability of Lon–mtHsp70 interaction. Through binding with Hsp60–mtHsp70 complex, increased mitochondrial Lon inhibits apoptosis and contributes to cell survival under environmental stress, by which they are likely to have important roles in a cytoprotective chaperone network (Figure 7).

By analyzing purified Lon-associated proteins from in-solution digestion shotgun proteomics, 76 candidate proteins of Lon-bound were identified (Supplementary Table S5). According to their nature and function, mitochondrial proteins of interest were classified into five functional categories: (1) mitochondrial chaperones, (2) cellular metabolism and energy, (3) Redox regulation, (4) cell death and survival, and (5) mtDNA stability (Table 1). From this list, we identified that NDUFS8, Hsp60, and mtHsp70 are binding partners of Lon, and they were validated by Co-IP and co-localization experiments, which is consistent with the finding that Lon interacts with NDUFS8 of mitochondrial complex I that is involved in the ROS generation induced by Lon.^11^ NDUFS8, also known as NADH-ubiquinone iron-sulfur (Fe-S) 23 kDa subunit, is a subunit of mitochondrial NADH oxidoreductase (Complex I) that functions in the transfer of electrons from NADH to the respiratory chain in the mitochondrial inner membrane. In addition, mitochondrial Fe-S proteins in complex I, II, and III, that is, SDHB (complex II) and Rieske (complex III), and COX 4–1 in complex IV are potential substrates of Lon protease.^16, 30, 31^ These results suggest that Lon may maintain Fe-S protein homeostasis of mitochondrial complexes I–IV and further energy generation. Indeed, yeast Lon regulates electron transport chain by degrading the subunits of complexes III–V in yeast.^31, 32^ Although Lon is a mitochondrial matrix protein, a number of proteins identified are located in the inner membrane, such as NDUFS8 and COX. Previous reports showed that yeast Lon interacts with prohibitin complex that could serve as a recruiter complex in the inner membrane to assist the quality control of these membrane proteins by Lon protease and to maintain the organization and stability of the mitochondrial nucleoids.^32, 33^

mtDNA nucleoid was postulated to be linked with the mitochondrial inner membrane.^34^ Lon is an DNA-binding protein that has been found to specifically bind sequences of DNA and has an important role in mtDNA maintenance and gene expression.^35, 36^ In the present study, we identified several Lon-associated proteins, for example, mtHSP70, Hsp60, and ATPB (Table 1), which are associated with mtDNA to form nucleoids.^26, 37^ Thereby, under the circumstance of Lon overexpression, it is conceivable that mitochondrial nucleoid proteins (Hsp60 and mtHSP70) would be stabilized on mtDNA, which could possibly increase the protection of mtDNA against oxidative damage.

We, for the first time, revealed the mechanism of how mitochondrial Lon regulates apoptosis. We identified Hsp60–mtHsp70 complex as a binding partner of Lon and found that the protein stability/level of Hsp60 and mtHsp70 depends on the level of Lon under oxidative stress. Most importantly, the ability of increased Lon-inhibited apoptosis is dependent on Hsp60. Thus we suggest that the mechanism underlying cell survival regulated by Lon is mediated by the maintenance of the stability of Hsp60 and mtHsp70 (Figure 7). Hsp60 exhibits both anti-apoptotic^25, 38^ and pro-apoptotic^35, 39, 40, 41^ functions depending on the context of cell type and condition.^29, 42, 43^ Mitochondrial Hsp60 inhibits apoptosis by increasing the stabilization of survivin, restraining p53 function,^25^ antagonizing cyclophilin D-dependent mitochondrial permeability transition,^44^ and preserving ATP generation of the complex IV.^43^ On the other hand, mitochondrial Hsp60 induces apoptosis by accelerating the maturation of pro-caspase-3.^35, 41^ The scenarios for the mechanism underlying Lon-enhanced cell survival through binding with Hsp60 and mtHsp70 are proposed. First, mitochondrial Lon stabilize Hsp60 and mtHsp70 to allow them to execute their anti-apoptotic function. Indeed, overexpression of Hsp60 and mtHsp70 was found in human tumor cells,^24, 45^ and they have been shown to antagonize p53 and abolish its apoptotic functions in cancer cells.^46^ Second, increased Lon stabilize the complex of Hsp60–mtHsp70–Lon to sequestrate Hsp60 in mitochondria, preventing it from the direct activation of pro-caspase-3 and cytoplasmic translocation that sensitizes cells to apoptosis.^47^ Third, to keep mitochondria integrity, Lon performs chaperone activity to cooperate with Hsp60–mtHsp70 complex and maintain protein homeostasis in mitochondria under stress, which is supported by mtHsp70 that may help Lon chaperone misfolded proteins to preserve mitochondria functions in yeast.^32, 48^ In short, the loss in the balance between Lon and Hsp60 within Lon–Hsp60–mtHsp70 complex will affect the activation of apoptosis and the cell survival. These HSP levels are elevated in various types of human tumors, which performs critical chaperone function to augment cell survival under stressful conditions.^19^

In summary, we identified and validated NDUFS8, Hsp60, and mtHsp70 as Lon-associated proteins through a proteomic approach that combines Co-IP and in-solution digestion. We next proved that the protein level of Hsp60 and mtHsp70 is dependent on Lon, and Lon-mediated apoptosis is controlled by the Hsp60–mtHsp70 complex. According to the Lon interactome, we suggest that Lon is a multifunctional protein and involves in the modulation of mitochondrial chaperones, cell death and survival, and mtDNA stability. Our studies will provide new insights into the role of Lon in mitochondrial biology exerted by its chaperone function and will allow us to understand how Lon overexpression promotes cell survival and tumorigenesis.

## Materials and Methods

### Cell culture, cell treatment, and retroviral infection

293, 293T, and FADU cells were cultured in medium containing DMEM (GIBCO, New York, NY, USA), supplemented with 5% FBS and 5% super calf serum and penicillin–streptomycin (50 U/ml, Sigma, St. Louis, MO, USA) in a 5% CO_2_/95% air atmosphere. Cultured cells were treated with H_2_O_2_ (Sigma-Aldrich, St. Louis, MO, USA) for 200 *μ*M at 37 °C, and the medium was changed to recover for the indicated period. Stable cell lines expressing tagged Lon or Lon-shRNA were generated by retroviral infection using pBabe or pMKO vector, respectively. Briefly, the plasmids, Lon-Myc-pBABE-puro for stable expression and Lon-Sh1/2-pMKO-puro for stable knockdown cells, along with packaging plasmid gag-pol and envelope plasmid VSV-G, were transfected into 293T cells by Lipofectamine 2000 (Invitrogen, Carlsbad, CA, USA). The cells were incubated for 24 h, and the medium was changed to remove remaining transfection reagent. Retroviral supernatant was collected at 24 and 48 h posttransfection and used to infect the target cells 293 for 48 h. Polybrene (hexadimethrine bromide) was added to the medium for improving infection efficacy. Puromycin (Sigma-Aldrich) was used to select the successfully infected cells at a final concentration of 2 *μ*g/ml, and the survived cells were collected to check the expression of human Lon by western blotting.

### Reagents and cell treatment

Cultured cells were treated with 200 *μ*M H_2_O_2_ (Sigma-Aldrich) for 1 or 4 h at 37 °C, and the medium was changed to recover for the indicated period. Sequences of the shRNA target sites are as shown below. Lon-shRNA: 5′-GAAAGUUCGUCUCGCCCAGCC-3′ (sh-1) or 5′-AGGAGCAGCUAAAGAUCAUCA-3′ (sh-2).^49^ For mitochondrial location, MitoTracker Red FM (Invitrogen) was added in DMEM at a final concentration of 500 nM for 30 min at 37 °C.

### Plasmid construction

Human Lon protease with Myc tag was cut by EcoRV (New England Biolabs, Hitchin, Hertfordshire, UK) from Lon-Myc-pcDNA3*β* and subcloned into pBabe-puro vector, which was cut by the same restriction enzyme. The plasmid was transformed into the competent ECOS 101 cells (Yeastern Biotech, Taipei, Taiwan). Positive transformants were inoculated into LB broth containing 50 mg/ml ampicillin for plasmid propagation. Plasmid was isolated and checked by EcoRV. The sequence of Lon-Myc-pBabe-puro was verified by DNA sequencing (Genomics BioSci&Tech. Co., Taipei, Taiwan). For construction of shRNA expression vectors, the retrovirus vector pMKO-puro^50^ was used. Sequences of the siRNA target sites for human Lon protease are as shown above.

### Co-immunoprecipitation (Co-IP)

Cell lysates from 293T, 293, or 293/Lon cells were incubated with control or indicated antibodies overnight at 4 °C. Anti-c-Myc Agarose Affinity Gel (Sigma-Aldrich) was used to pull-down the protein complex. Immunocomplex were pelleted by incubation with protein G-agarose (GE Healthcare, Uppsala, Sweden) for 3 h at 4 °C with slow agitation and centrifugation for 15 s. The pellets were washed three times with NETN (20 mM Tris (pH 8.0), 1 mM EDTA, 150 mM NaCl, 0.5% Nonidet P-40 (NP-40)) containing protease inhibitor cocktail (Roche, Mannheim, Germany) buffer and could be examined for binding partners by western blotting. For shotgun proteomic analysis, immunocomplex were eluted by 6 M urea in 100 mM Tris buffer with slow agitation for 10 min at room temperature.

### In-gel digestion

IP eluate was separated by 10% SDS-PAGE and stained by SYPRO Ruby (Invitrogen). The protein bands excised from SDS-PAGE were reduced with 50 mM dithiothreitol (DTT, J.T. Baker, Phillipsburg, NJ, USA) in 25 mM ammonium bicarbonate (pH 8.5) at 37 °C for 1 h and alkylated by 100 mM iodoacetamide (IAA, Sigma-Aldrich) in 25 mM ammonium bicarbonate in the dark at room temperature for 1 h. The gels were destained with 5% ACN (acetonitrile) in 25 mM ammonium bicarbonate. After dehydrated in 100% ACN and dried in a SpeedVac (Thermo Fisher Scientific Inc., Waltham, MA, USA), the gel pieces were rehydrated with 0.0225 *μ*g trypsin (Promega, Madison, WI, USA) in 25 mM ammonium bicarbonate and incubated overnight at 37 °C with slow agitation. Crashing the gels before tryptic peptides were extracted from the gels with sonication in 50% ACN/5% TFA (trifluoroacetic acid). Finally, the extractions were dried in a SpeedVac and dissolved with 10 *μ*l 0.1% formic acid. For mass spectrometry analysis, sample was desalted using ZipTip (Millipore, Billerica, MA, USA) according to the manufacturer's instructions.

### In solution digestion

The protein sample was re-suspended in the 6 M urea/100 mM Tris buffer. 200 mM dithiothreitol (DTT, J.T. Baker) was added to the protein solution and incubated in the dark for 1 h at room temperature. After incubation, proteins were alkylated by 200 mM IAA (Sigma-Aldrich) for 1 h at room temperature in the dark. The sample was then diluted six times with 25 mM ammonium bicarbonate reducing the concentration of urea to 0.6 M and digested with sequencing grade modified trypsin (1/50 amount of the protein, Promega) at 37 °C overnight. The tryptic digestion was quenched by adding 20% TFA to pH <3. The resulting peptides were desalted and concentrated using SDB-XC column (3M, St. Paul, MN, USA), which was a poly (styrenedivinylbenzene) copolymer used as a reversed-phase sorbent for solid-phase extraction, then dried by SpeedVac, and re-dissolved in 10 *μ*l 0.1% formic acid. For nanoLC-MS/MS (nanoflow liquid chromatography tandem mass spectrometry) analysis, sample was desalted using ZipTip (Millipore) according to the manufacturer's instructions.

### Method for removing detergents from IP eluate

IP eluate containing NP-40 were loaded (total volume <200 *μ*l) in Vivaspin 10 kDa (GE Healthcare) that had been rinsed by doubly distilled water and then centrifuged by 13 000 r.p.m. for 30 min at room temperature. After centrifugation, the remaining solution in concentrator was re-suspended with 6 M urea/100 mM Tris buffer and centrifuged at 13 000 r.p.m. for 30 min at room temperature. Finally, the concentrated proteins were washed by 8 M urea/100 mM Tris buffer to remove detergents retaining in IP eluate.^51^

### nanoLC-MS/MS analysis

The peptides mixtures were analyzed by online nanoLC-MS/MS on a nanoAcquity system (Waters, Milford, MA, USA) coupled to an LTQ-Orbitrap Velos hybrid mass spectrometer (Thermo Fisher Scientific Inc.) equipped with a PicoView nanospray interface (New Objective, Woburn, MA, USA). Peptide mixtures were loaded onto a 75 *μ*m × 250 mm nanoACQUITY UPLC BEH130 column packed with C18 resin (Waters). The effluent from the HPLC column was directly electrosprayed into the mass spectrometer. The LTQ Orbitrap Velos instrument was operated in a data-dependent mode to automatically switch between full-scan MS and MS/MS acquisition. Instrument control was through Tune 2.6.0 and Xcalibur 2.1. For the CID-MS/MS top20 method, full-scan MS spectra (*m/z* 350–1600) were acquired in the Orbitrap analyzer after accumulation to a target value of 1e6 in the linear ion trap. Resolution in the Orbitrap system was set to *R*=60 000 (all Orbitrap system resolution values are given at *m/z* 400). The 20 most intense peptide ions with charge states ≥2 were sequentially isolated to a target value of 5000 and fragmented in the high-pressure linear ion trap by low-energy CID with normalized collision energy of 35%. The resulting fragment ions were scanned out in the low-pressure ion trap at the normal scan rate and recorded with the secondary electron multipliers.

### Data processing and bioinformatics

All MS and MS/MS raw data were processed by Raw2MSM and searched against SwissProt database using the Mascot Daemon 2.2 server (Matrix Science, Boston, MA, USA). Search criteria used were as follows: the algorithm was set to use *Homo sapiens* as taxonomy and trypsin as the enzyme; variable modifications set as carbamidomethyl (Cys) and oxidation (Met); up to two missed cleavages allowed; and mass accuracy of 10 p.p.m. for the parent ion and 0.60 Da for the fragment ions. Protein hits were taken as identified if the mascot score exceeded the threshold of *P*<0.05, indicating identification at the 95% confidence level. Proteins identified by shotgun proteomic were analyzed further by using the network building tool MetaCore (GeneGo) for pathway analysis and IPA to identify the functional groups of the identified proteins.

### Antibodies

Antibodies to human Lon was produced as described previously.^52^ Antibodies used in this study were purchased as indicated: antibody to Myc (9E10) from Millipore; HSP60 (ab46798) and COX4 (ab16056) from Abcam (Cambridge, MA, USA); cleaved-caspase 3, cleaved-PARP, and phosphorylated p53^Ser46^ (no. 2521)^53^ from Cell Signaling Technology (Beverly, MA, USA); Bax from Santa Cruz Biotechnology, Inc. (Santa Cruz, CA, USA); caspase 3 (IMG-144A) from IMGENX; mtHSP70 from Thermo Fisher Scientific Inc.; and GAPDH and beta-actin from GenTex (Hsinchu, Taiwan).

### Western blotting analysis

The cells were harvested by trypsinization and lysed with NETN buffer (20 mM Tris (pH 8.0), 1 mM EDTA, 150 mM NaCl, 0.5% Nonidet P-40 (NP-40)) containing protease inhibitor cocktail (Roche). The cell lysates were then centrifuged at 10 000 × *g* at 4 °C to obtain solubilized cellular proteins. Protein was quantified with a bicinchoninic acid protein assay (Pierce, Rockford, IL, USA) according to the manufacturer's instructions. Proteins were separated by 8% or 10% or 12% SDS-PAGE and electrotransfered to a polyvinylidene fluoride membrane. Blots were probed with primary antibodies, followed by HRP-conjugated goat anti-rabbit IgG (1 : 5000, v/v) (Zymed, South San Francisco, CA, USA) or HRP-conjugated goat anti-mouse IgG (1 : 5000, v/v) (Zymed). After washing with phosphate-buffered saline (PBS) containing 0.5% Tween-20, peroxidase activity was assessed using enhanced chemiluminescence (PerkinElmer Life Science, Boston, MA, USA). For an internal control, the same membrane was re-probed with a monoclonal antibody directed against *β*-actin (1 : 10 000, v/v) or GAPDH (1 : 5000). The intensities of the reaction bands were analyzed with the Image Gauge System (Fuji, Tokyo, Japan).

### Immunofluorescence

Cells were plated on glass coverslips placed in a 12-well culture dish. When cells had attached to the surface and spread well, they were washed with cold PBS and then fixed with precold methanol/acetone (1 : 1, v/v) mixture for 15 min at room temperature. Fixed cells were washed with PBS and permeabilized with 0.5% (v/v) Triton X-100 in PBS for 15 min at room temperature. Cells on coverslips were incubated with the indicated antibodies: anti-Lon (1 : 400), anti-Myc (1 : 200), anti-Hsp60 (1 : 200), and anti-mtHsp70 (1 : 200) overnight at 4 °C. The following day, fixed cells were washed three times with 0.5% Triton X-100 in PBS and incubated with Alexa 488-conjugated and Alexa-594 conjugated anti-mouse or anti-rabbit secondary antibodies. Finally, coverslips were mounted by ProLong Gold Antifade Reagent with DAPI (Invitrogen) at room temperature for 10 min. Fluorescent images were acquired by Olympus (Tokyo, Japan) BX51 and images were processed using the Image J software (National Institutes of Health, Bethesda, MD, USA). For confocal immunofluorescence, the cells were incubated with anti-Lon antibody (1 : 200), anti-NDUFS8 (1 : 200), or anti-mtHsp70 (1 : 200) for 1 h at 37 °C, and then with goat anti-mouse DyLight fluorescence-conjugated secondary antibody for 1 h at RT. The above experimental results were analyzed by a Leica (Wetzlar, Germany) TCS SP5 II confocal fluorescence microscopy.

### Cell viability analysis

Cell viability analysis was performed by cell number counting. For cell number counting, cells were seeded on six-well plastic dishes at a concentration of 1 × 10^5^ per well. Prior to the experiment, 60–70% confluence cells were washed twice with PBS and treated with UV or not. Cells were then stained with trypan blue, and cell numbers were counted under an inverted microscope (Leica). Data are presented as the mean±S.D. of three replicates from six separate experiments.

### Apoptosis assay

Apoptosis was analyzed by TUNEL staining or Annexin V/PI staining. Cell apoptosis was detected by TUNEL assay according to the manufacturer's instructions (TaKaRa BIO, Shiga, Japan) and was performed as described previously.^11, 54^ Apoptotic cells also were analyzed by flow cytometry after Annexin V/PI staining using the FITC Annexin V Apoptosis Detection Kit I (BD Biosciences, San Diego, CA, USA) according to the manufacturer's protocols.

### Statistical methods

Parametric Student's *t*-test was used to judge the significance of difference between conditions of interest. In general, a *P*-value of <0.05 was considered as statistically significant (Student's *t*-test, *P<0.05, **P<0.01, and ***P<0.001).

## Figures and Tables

**Table 1:** Classification of Lon-associated mitochondrial proteins of interest into functional categories in this study

**Functional categories**	**Biological process associated**
*Mitochondrial chaperones*	
Stress-70 protein, mitochondrial (mtHSP70)	Protein folding in the matrix
60 kDa heat shock protein, mitochondrial (HSP60)	Protein folding in the matrix
10 kDa heat-shock protein, mitochondrial (HSP10)	Protein folding in the matrix
78 kDa glucose-regulated protein (GRP78)	Unfolded protein response
	
*Cellular metabolism and energy*	
Dihydrolipoamide dehydrogenase (DLDH)	Pyruvate oxidation
Methylmalonic aciduria and homocystinuria type D protein, mitochondrial (MMAD)	Vitamin B12 metabolism and fatty acid oxidation
ATP synthase subunit beta (ATPB)	Complex V in respiratory chain
NADH dehydrogenase [ubiquinone] iron-sulfur protein 8 (NDUFS8)	Complex I in respiratory chain
Coenzyme Q biosynthesis monooxygenase (CoQ6)	Respiratory chain
	
*Redox regulation*	
Thioredoxin2 (THIO2)	Antioxidant
Peroxiredoxin-1 (PRDX1)	Antioxidant
Peroxiredoxin-2 (PRDX2)	Antioxidant
	
*Cell death and survival*	
60 kDa heat shock protein, mitochondrial (HSP60)	Apoptosis regulation
Stress-70 protein, mitochondrial (mtHSP70)	Apoptosis regulation
Caspase 14	AIF binding protein
14-3-3	Target mitochondrial apoptotic proteins
Annexin A2	Components of ceramide platform
	
*Mitochondrial DNA stability*	
Myosin 9	Mitochondrial DNA maintenance
Myosin 10	Mitochondrial DNA maintenance
Actin	Mitochondrial DNA maintenance
Stress-70 protein, mitochondrial (mtHSP70)	Mitochondrial nucleoid protein
60 kDa heat-shock protein, mitochondrial (HSP60)	Mitochondrial nucleoid protein
ATP synthase subunit beta (ATPB)	Mitochondrial nucleoid protein

## Abbreviations

ATP: adenosine-5′-triphosphate
Co-IP: co-immunoprecipitation
COX: cytochrome *c* oxidase
H_2_O_2_: hydrogen peroxide
Hsp60: heat-shock protein 60
IPA: Ingenuity Pathways Analysis
mtDNA: mitochondrial DNA
NDUFS8: NADH dehydrogenase [ubiquinone] iron-sulfur protein 8
nanoLC-MS/MS: nanoflow liquid chromatography tandem mass spectrometry
TUNEL: terminal deoxynucleotidyl transferase-mediated dUTP nick-end labeling
