# Supplementary material for: Mitochondrial Lon regulates apoptosis through the association with Hsp60–mtHsp70 complex
Source: Cell Death Dis. 2015 Feb 12;6(2):e1642–. doi: 10.1038/cddis.2015.9 (PMC4669791; doi:10.1038/cddis.2015.9)
Supplement: Supplementary Tables [file cddis20159x1.doc]

**Supplemental Table 1 Chaperone Lon-associated proteins identified by in-gel-digestion and mass spectrometry**

|  | **Accession Number (Uniprot ID)** | **Protein name** | **Mascot Socre** | **Molecular Mass (Daltons)** | **Matching Peptides** |
| --- | --- | --- | --- | --- | --- |
| 1 | LONM_HUMAN | Lon protease homolog, mitochondrial | 2845 | 106422 | 51 |
| 2 | HSP71_HUMAN | Heat shock 70 kDa protein 1A/1B | 1038 | 70009 | 22 |
| 3 | MYH9_HUMAN | Myosin-9 | 925 | 226392 | 23 |
| 4 | BICD2_HUMAN | Protein bicaudal D homolog 2 | 725 | 93476 | 28 |
| 5 | HS71L_HUMAN | Heat shock 70 kDa protein 1-like | 674 | 70331 | 13 |
| 6 | HSP7C_HUMAN | Heat shock cognate 71 kDa protein | 522 | 70854 | 13 |
| 7 | HSP76_HUMAN | Heat shock 70 kDa protein 6 | 489 | 70984 | 9 |
| 8 | M21D2_HUMAN | Protein MB21D2 | 387 | 55764 | 16 |
| 9 | HSP77_HUMAN | Putative heat shock 70 kDa protein 7 | 371 | 40220 | 6 |
| 10 | GRP75_HUMAN | Stress-70 protein, mitochondrial | 354 | 73635 | 12 |
| 11 | HSP72_HUMAN | Heat shock-related 70 kDa protein 2 | 284 | 69978 | 5 |
| 12 | DESP_HUMAN | Desmoplakin | 255 | 331569 | 17 |
| 13 | TBA1A_HUMAN | Tubulin alpha-1A chain | 145 | 50104 | 5 |
| 14 | MYH10_HUMAN | Myosin-10 | 144 | 228858 | 4 |
| 15 | TBB5_HUMAN | Tubulin beta chain | 141 | 49639 | 4 |
| 16 | ACTA_HUMAN | Actin, aortic smooth muscle | 132 | 41982 | 3 |
| 17 | TBB4A_HUMAN | Tubulin beta- 4A chain | 128 | 49554 | 4 |
| 18 | XRCC6_HUMAN | X-ray repair cross-complementing protein 6 | 125 | 69799 | 6 |
| 19 | DCD_HUMAN | Dermcidin | 123 | 11277 | 2 |
| 20 | TBB2A_HUMAN | Tubulin beta-2A chain | 122 | 49875 | 3 |
| 21 | ACTBL_HUMAN | Beta-actin-like protein 2 | 115 | 41976 | 2 |
| 22 | HNRPU_HUMAN | Heterogeneous nuclear ribonucleoprotein U | 105 | 90528 | 5 |
| 23 | HORN_HUMAN | Hornerin | 104 | 282228 | 1 |
| 24 | BICD1_HUMAN | Protein bicaudal D homolog 1 | 101 | 110682 | 5 |
| 25 | ACTBM_HUMAN | Putative beta-actin-like protein 3 | 98 | 41989 | 1 |
| 26 | TBA3C_HUMAN | Tubulin alpha-3C/D chain | 82 | 49928 | 3 |
| 27 | TBA3E_HUMAN | Tubulin alpha-3E chain | 77 | 49827 | 2 |
| 28 | GFAP_HUMAN | Glial fibrillary acidic protein | 76 | 49850 | 2 |
| 29 | NFH_HUMAN | Neurofilament heavy polypeptide | 76 | 112411 | 2 |
| 30 | OPTN_HUMAN | Optineurin | 76 | 65880 | 2 |
| 31 | PIP_HUMAN | Prolactin-inducible protein | 76 | 16562 | 1 |
| 32 | TBB8_HUMAN | Tubulin beta-8 chain | 73 | 49744 | 2 |
| 33 | GRP78_HUMAN | 78 kDa glucose-regulated protein | 66 | 72288 | 1 |
| 34 | TBA4A_HUMAN | Tubulin alpha-4A chain | 58 | 49892 | 2 |
| 35 | FLNA_HUMAN | Filamin-A | 56 | 280564 | 2 |
| 36 | PLAK_HUMAN | Junction plakoglobin | 56 | 81693 | 1 |
| 37 | TBB1_HUMAN | Tubulin beta-1 chain | 55 | 50295 | 1 |
| 38 | MYH14_HUMAN | Myosin-14 | 54 | 227732 | 5 |
| 39 | CLH1_HUMAN | Clathrin heavy chain 1 | 51 | 191493 | 1 |
| 40 | TBA8_HUMAN | Tubulin alpha-8 chain | 50 | 50062 | 2 |
| 41 | SYK_HUMAN | Lysine--tRNA ligase | 46 | 68005 | 3 |
| 42 | IGHG2_HUMAN | Ig gamma-2 chain C region | 44 | 35,901 | 1 |
| 43 | PSMD1_HUMAN | 26S proteasome non-ATPase regulatory subunit 1 | 35 | 105769 | 2 |
| 44 | PCD18_HUMAN | Protocadherin-18 | 34 | 126071 | 1 |
| 45 | PCMD2_HUMAN | Protein-L-isoaspartate O-methyltransferase domain-containing protein 2 | 33 | 41046 | 3 |
| 46 | WIPI2_HUMAN | WD repeat domain phosphoinositide-interacting protein 2 | 29 | 49377 | 1 |
| 47 | AKA11_HUMAN | A-kinase anchor protein 11 | 24 | 210380 | 1 |
| 48 | ABCA8_HUMAN | ATP-binding cassette sub-family A member 8 | 24 | 179130 | 1 |

**Supplemental Table 2 Chaperone Lon-associated proteins identified by in-solution-digestion and mass spectrometry**

|  | **Accession Number (Uniprot ID)** | **Protein name** | **Mascot Socre** | **Molecular Mass (Daltons)** | **pI** | **Sequence Coverage (%)** | **Matching Peptides** |
| --- | --- | --- | --- | --- | --- | --- | --- |
| 1 | DESP_HUMAN | Desmoplakin | 971 | 331569 | 6.44 | 21.7 | 71 |
| 2 | VIME_HUMAN | Vimentin | 775 | 53619 | 5.06 | 71.2 | 39 |
| 3 | PLAK_HUMAN | Junction plakoglobin | 395 | 81693 | 5.75 | 31.7 | 26 |
| 4 | MYH9_HUMAN | Myosin-9 | 376 | 226392 | 5.5 | 14.3 | 26 |
| 5 | DSG1_HUMAN | Desmoglein-1 | 345 | 113676 | 4.9 | 24.6 | 23 |
| 6 | HSP71_HUMAN | Heat shock 70 kDa protein 1A/1B | 312 | 70009 | 5.48 | 29.5 | 18 |
| 7 | NUCL_HUMAN | Nucleolin | 249 | 76568 | 4.6 | 23.4 | 14 |
| 8 | DLDH_HUMAN | Dihydrolipoyl dehydrogenase, mitochondrial | 239 | 54143 | 7.95 | 29.3 | 14 |
| 9 | TPM1_HUMAN | Tropomyosin alpha-1 chain | 234 | 32689 | 4.69 | 47.2 | 19 |
| 10 | HNRPC_HUMAN | Heterogeneous nuclear ribonucleoproteins C1/C2 | 230 | 33650 | 4.95 | 32 | 11 |
| 11 | RLA2_HUMAN | 60S acidic ribosomal protein P2 | 226 | 11658 | 4.42 | 73.9 | 7 |
| 12 | TPM3_HUMAN | Tropomyosin alpha-3 chain | 218 | 32799 | 4.68 | 41.9 | 18 |
| 13 | CH60_HUMAN | 60 kDa heat shock protein, mitochondrial | 201 | 61016 | 5.7 | 18.2 | 8 |
| 14 | H2B1B_HUMAN | Histone H2B type 1-B | 201 | 13942 | 10.31 | 31.7 | 6 |
| 15 | LONM_HUMAN | Lon protease homolog, mitochondrial | 199 | 106422 | 6.01 | 15.2 | 14 |
| 16 | HSP76_HUMAN | Heat shock 70 kDa protein 6 | 196 | 70984 | 5.81 | 12.3 | 8 |
| 17 | ROA1_HUMAN | Heterogeneous nuclear ribonucleoprotein A1 | 189 | 38723 | 9.17 | 23.4 | 9 |
| 18 | TPIS_HUMAN | Triosephosphate isomerase | 189 | 30772 | 5.65 | 30.8 | 7 |
| 19 | HSP7C_HUMAN | Heat shock cognate 71 kDa protein | 181 | 70854 | 5.37 | 22.4 | 13 |
| 20 | H13_HUMAN | Histone H1.3 | 176 | 22336 | 11.02 | 22.6 | 21 |
| 21 | RO52_HUMAN | E3 ubiquitin-protein ligase TRIM21 | 174 | 54135 | 5.98 | 36.8 | 17 |
| 22 | CASPE_HUMAN | Caspase-14 | 166 | 27662 | 5.44 | 25.6 | 9 |
| 23 | TPM4_HUMAN | Tropomyosin alpha-4 chain | 162 | 28504 | 4.67 | 48.8 | 16 |
| 24 | NPM_HUMAN | Nucleophosmin | 157 | 32555 | 4.64 | 36.1 | 11 |
| 25 | HNRPK_HUMAN | Heterogeneous nuclear ribonucleoprotein K | 156 | 50944 | 5.39 | 17.3 | 6 |
| 26 | HS71L_HUMAN | Heat shock 70 kDa protein 1-like | 152 | 70331 | 5.76 | 19.8 | 11 |
| 27 | GRP78_HUMAN | 78 kDa glucose-regulated protein | 151 | 72288 | 5.07 | 26.6 | 16 |
| 28 | TPM2_HUMAN | Tropomyosin beta chain | 143 | 32831 | 4.66 | 31.3 | 13 |
| 29 | CBX3_HUMAN | Chromobox protein homolog 3 | 131 | 20798 | 5.23 | 26.8 | 4 |
| 30 | KPRP_HUMAN | Keratinocyte proline-rich protein | 126 | 64093 | 8.72 | 22.8 | 16 |
| 31 | SPB12_HUMAN | Serpin B12 | 122 | 46247 | 5.36 | 16.5 | 8 |
| 32 | YBOX1_HUMAN | Nuclease-sensitive element-binding protein 1 | 121 | 35903 | 9.87 | 15.7 | 4 |
| 33 | LMNA_HUMAN | Prelamin-A/C | 106 | 74095 | 6.57 | 3.2 | 3 |
| 34 | CALL5_HUMAN | Calmodulin-like protein 5 | 105 | 15883 | 4.34 | 21.9 | 2 |
| 35 | EF1D_HUMAN | Elongation factor 1-delta | 104 | 31103 | 4.9 | 16.4 | 4 |
| 36 | GOGA1_HUMAN | Golgin subfamily A member 1 | 103 | 88130 | 5.24 | 15.6 | 12 |
| 37 | ARGI1_HUMAN | Arginase-1 | 99 | 34713 | 6.72 | 20.8 | 7 |
| 38 | HNRH1_HUMAN | Heterogeneous nuclear ribonucleoprotein H | 97 | 49198 | 5.89 | 15.1 | 5 |
| 39 | NFH_HUMAN | Neurofilament heavy polypeptide | 92 | 112411 | 5.99 | 2.9 | 8 |
| 40 | RCN2_HUMAN | Reticulocalbin-2 | 92 | 36854 | 4.26 | 7.3 | 3 |
| 41 | CALR_HUMAN | Calreticulin | 90 | 48112 | 4.29 | 21.6 | 8 |
| 42 | MMAD_HUMAN | Methylmalonic aciduria and homocystinuria type D protein, mitochondrial | 90 | 32919 | 5.16 | 15.9 | 3 |
| 43 | TGM3_HUMAN | Protein-glutamine gamma-glutamyltransferase E | 88 | 76584 | 5.62 | 7.2 | 7 |
| 44 | ACTB_HUMAN | Actin, cytoplasmic 1 | 87 | 41710 | 5.29 | 25.1 | 8 |
| 45 | MYH10_HUMAN | Myosin-10 | 83 | 228858 | 5.44 | 5.3 | 9 |
| 46 | CALM_HUMAN | Calmodulin | 79 | 16827 | 4.09 | 37.6 | 7 |
| 47 | DSC1_HUMAN | Desmocollin-1 | 76 | 99924 | 5.25 | 6 | 5 |
| 48 | CBX1_HUMAN | Chromobox protein homolog 1 | 75 | 21405 | 4.85 | 14.6 | 2 |
| 49 | PPBI_HUMAN | Intestinal-type alkaline phosphatase | 73 | 56776 | 5.53 | 1.9 | 1 |
| 50 | NACA_HUMAN | Nascent polypeptide-associated complex subunit alpha | 73 | 23370 | 4.52 | 7 | 1 |
| 51 | HNRPF_HUMAN | Heterogeneous nuclear ribonucleoprotein F | 71 | 45643 | 5.38 | 12 | 4 |
| 52 | SET_HUMAN | Protein SET | 71 | 33469 | 4.23 | 15.5 | 5 |
| 53 | DESM_HUMAN | Desmin | 69 | 53503 | 5.21 | 13.8 | 7 |
| 54 | S10A9_HUMAN | Protein S100-A9 | 69 | 13234 | 5.71 | 39.5 | 4 |
| 55 | PPBN_HUMAN | Alkaline phosphatase, placental-like | 68 | 57341 | 5.9 | 3 | 2 |
| 56 | G3P_HUMAN | Glyceraldehyde-3-phosphate dehydrogenase | 68 | 36030 | 8.57 | 22.7 | 6 |
| 57 | PIP_HUMAN | Prolactin-inducible protein | 67 | 16562 | 8.26 | 11 | 3 |
| 58 | TBA1A_HUMAN | Tubulin alpha-1A chain | 64 | 50104 | 4.94 | 7.3 | 2 |
| 59 | TFG_HUMAN | Protein TFG | 63 | 43421 | 4.94 | 9.3 | 3 |
| 60 | CATD_HUMAN | Cathepsin D | 60 | 44524 | 6.1 | 7 | 3 |
| 61 | THIO_HUMAN | Thioredoxin | 60 | 11730 | 4.82 | 32.4 | 3 |
| 62 | CATA_HUMAN | Catalase | 59 | 59719 | 6.9 | 5.9 | 2 |
| 63 | H11_HUMAN | Histone H1.1 | 55 | 21829 | 10.99 | 14 | 4 |
| 64 | PRDX1_HUMAN | Peroxiredoxin-1 | 55 | 22096 | 8.27 | 21.6 | 3 |
| 65 | ENOA_HUMAN | Alpha-enolase | 53 | 47139 | 7.01 | 6.7 | 2 |
| 66 | TBB2A_HUMAN | Tubulin beta-2A chain | 53 | 49875 | 4.78 | 13.7 | 5 |
| 67 | TBB2C_HUMAN | Tubulin beta-2C chain | 53 | 49799 | 4.79 | 13.7 | 5 |
| 68 | TBB3_HUMAN | Tubulin beta-3 chain | 53 | 50400 | 4.83 | 11.8 | 4 |
| 69 | CLCF1_HUMAN | Cardiotrophin-like cytokine factor 1 | 52 | 25160 | 8.68 | 3.6 | 2 |
| 70 | RS27A_HUMAN | Ubiquitin-40S ribosomal protein S27a | 52 | 17953 | 9.68 | 16 | 2 |
| 71 | CTNB1_HUMAN | Catenin beta-1 | 51 | 85442 | 5.53 | 4.6 | 3 |
| 72 | RBBP4_HUMAN | Histone-binding protein RBBP4 | 51 | 47626 | 4.74 | 7.8 | 4 |
| 73 | RLA1_HUMAN | 60S acidic ribosomal protein P1 | 50 | 11507 | 4.26 | 14 | 1 |
| 74 | MYL3_HUMAN | Myosin light chain 3 | 49 | 21918 | 5.03 | 12.3 | 4 |
| 75 | HNRL1_HUMAN | Heterogeneous nuclear ribonucleoprotein U-like protein 1 | 46 | 95679 | 6.49 | 5.4 | 4 |
| 76 | NONO_HUMAN | Non-POU domain-containing octamer-binding protein | 46 | 54197 | 9.01 | 5.9 | 4 |
| 77 | GDE_HUMAN | Glycogen debranching enzyme | 45 | 174652 | 6.31 | 0.9 | 1 |
| 78 | RL23A_HUMAN | 60S ribosomal protein L23a | 44 | 17684 | 10.44 | 21.8 | 3 |
| 79 | RCN1_HUMAN | Reticulocalbin-1 | 44 | 38866 | 4.86 | 15.4 | 5 |
| 80 | WDR91_HUMAN | WD repeat-containing protein 91 | 44 | 83292 | 6.15 | 0.9 | 1 |
| 81 | COF1_HUMAN | Cofilin-1 | 43 | 18491 | 8.22 | 8.4 | 1 |
| 82 | RL29_HUMAN | 60S ribosomal protein L29 | 42 | 17741 | 11.66 | 13.2 | 2 |
| 83 | AINX_HUMAN | Alpha-internexin | 41 | 55357 | 5.34 | 4.4 | 3 |
| 84 | NFL_HUMAN | Neurofilament light polypeptide | 41 | 61479 | 4.64 | 4.2 | 2 |
| 85 | NFM_HUMAN | Neurofilament medium polypeptide | 41 | 102411 | 4.9 | 4 | 4 |
| 86 | SRSF6_HUMAN | Serine/arginine-rich splicing factor 6 | 41 | 39563 | 11.42 | 8.7 | 4 |
| 87 | LEG7_HUMAN | Galectin-7 | 40 | 15066 | 7.03 | 27.2 | 3 |
| 88 | H4_HUMAN | Histone H4 | 40 | 11360 | 11.36 | 17.5 | 2 |
| 89 | IKIP_HUMAN | Inhibitor of nuclear factor kappa-B kinase-interacting protein | 40 | 39285 | 9.21 | 3.4 | 1 |
| 90 | NP1L1_HUMAN | Nucleosome assembly protein 1-like 1 | 39 | 45346 | 4.36 | 7.9 | 2 |
| 91 | HNRPU_HUMAN | Heterogeneous nuclear ribonucleoprotein U | 38 | 90528 | 5.76 | 4.1 | 3 |
| 92 | MARCS_HUMAN | Myristoylated alanine-rich C-kinase substrate | 38 | 31536 | 4.47 | 5.7 | 1 |
| 93 | GGCT_HUMAN | Gamma-glutamylcyclotransferase | 37 | 20994 | 5.07 | 11.7 | 3 |
| 94 | S10AE_HUMAN | Protein S100-A14 | 36 | 11655 | 5.16 | 10.6 | 1 |
| 95 | C1QBP_HUMAN | Complement component 1 Q subcomponent-binding protein, mitochondrial | 35 | 31343 | 4.74 | 5 | 1 |
| 96 | MDC1_HUMAN | Mediator of DNA damage checkpoint protein 1 | 35 | 226529 | 5.35 | 1.4 | 2 |
| 97 | S10A8_HUMAN | Protein S100-A8 OS=Homo sapiens GN=S100A8 PE=1 SV=1 | 34 | 10828 | 6.51 | 19.4 | 3 |
| 98 | SG1D2_HUMAN | Secretoglobin family 1D member 2 | 34 | 9918 | 8.58 | 10 | 1 |
| 99 | STRN4_HUMAN | Striatin-4 | 34 | 80546 | 5.21 | 1.2 | 1 |
| 100 | LAMB1_HUMAN | Laminin subunit beta-1 | 33 | 197909 | 4.83 | 0.9 | 2 |
| 101 | LAMB2_HUMAN | Laminin subunit beta-2 | 33 | 195854 | 6.07 | 0.8 | 2 |
| 102 | TRIPB_HUMAN | Thyroid receptor-interacting protein 11 | 33 | 227447 | 5.18 | 0.7 | 2 |
| 103 | URB2_HUMAN | Unhealthy ribosome biogenesis protein 2 homolog | 33 | 170435 | 6.97 | 1 | 2 |
| 104 | MIF_HUMAN | Macrophage migration inhibitory factor | 32 | 12468 | 7.74 | 7.8 | 1 |
| 105 | PTMS_HUMAN | Parathymosin | 32 | 11523 | 4.14 | 10.8 | 1 |
| 106 | TYB10_HUMAN | Thymosin beta-10 | 32 | 5023 | 5.31 | 31.8 | 1 |
| 107 | RL19_HUMAN | 60S ribosomal protein L19 | 31 | 23451 | 11.48 | 9.2 | 2 |
| 108 | EF1A1_HUMAN | Elongation factor 1-alpha 1 | 31 | 50109 | 9.1 | 2.2 | 1 |
| 109 | H1X_HUMAN | Histone H1x | 31 | 22474 | 10.76 | 4.7 | 1 |
| 110 | P2RX3_HUMAN | P2X purinoceptor 3 | 31 | 44260 | 7.88 | 2 | 1 |
| 111 | SF3A3_HUMAN | Splicing factor 3A subunit 3 | 31 | 58812 | 5.27 | 2.6 | 2 |
| 112 | XPO2_HUMAN | Exportin-2 | 30 | 110346 | 5.51 | 3.2 | 2 |
| 113 | RANG_HUMAN | Ran-specific GTPase-activating protein | 30 | 23296 | 5.19 | 22.4 | 3 |
| 114 | COQ6_HUMAN | Ubiquinone biosynthesis monooxygenase COQ6 | 30 | 50838 | 6.81 | 4.5 | 2 |
| 115 | DBPA_HUMAN | DNA-binding protein A | 29 | 40066 | 9.77 | 6.7 | 3 |
| 116 | HSPB1_HUMAN | Heat shock protein beta-1 | 29 | 22768 | 5.98 | 16.6 | 4 |
| 117 | TMF1_HUMAN | TATA element modulatory factor | 29 | 122767 | 4.88 | 3.3 | 3 |
| 118 | 1433B_HUMAN | 14-3-3 protein beta/alpha | 28 | 28065 | 4.76 | 8.9 | 2 |
| 119 | 1433E_HUMAN | 14-3-3 protein epsilon | 28 | 29155 | 4.63 | 7.8 | 2 |
| 120 | 1433G_HUMAN | 14-3-3 protein gamma | 28 | 28285 | 4.8 | 7.3 | 2 |
| 121 | 1433S_HUMAN | 14-3-3 protein sigma | 28 | 27757 | 4.68 | 8.5 | 2 |
| 122 | 1433T_HUMAN | 14-3-3 protein theta | 28 | 27747 | 4.68 | 7.3 | 2 |
| 123 | 1433Z_HUMAN | 14-3-3 protein zeta/delta | 28 | 27728 | 4.73 | 8.2 | 3 |
| 124 | RL32_HUMAN | 60S ribosomal protein L32 | 28 | 15850 | 11.32 | 17 | 2 |
| 125 | NDUFS8_HUMAN | NADH dehydrogenase [ubiquinone] iron-sulfur protein 8, mitochondrial | 28 | 23690 | 6 | 5.2 | 1 |
| 126 | SODC_HUMAN | Superoxide dismutase [Cu-Zn] | 28 | 15926 | 5.7 | 26 | 2 |
| 127 | IF2A_HUMAN | Eukaryotic translation initiation factor 2 subunit 1 | 27 | 36089 | 5.02 | 7.9 | 2 |
| 128 | PYGL_HUMAN | Glycogen phosphorylase, liver form | 27 | 97087 | 6.71 | 2.6 | 2 |
| 129 | CALU_HUMAN | Calumenin | 26 | 37084 | 4.47 | 14.3 | 4 |
| 130 | ZFY27_HUMAN | Protrudin | 26 | 45814 | 5.09 | 1.5 | 1 |
| 131 | CM035_HUMAN | Putative uncharacterized protein C13orf35 | 26 | 13408 | 8.66 | 8.3 | 2 |
| 132 | ANXA2_HUMAN | Annexin A2 | 25 | 38580 | 7.57 | 17.7 | 5 |
| 133 | ATPB_HUMAN | ATP synthase subunit beta, mitochondrial | 25 | 56525 | 5.26 | 2.1 | 1 |
| 134 | DJC15_HUMAN | DnaJ homolog subfamily C member 15 | 25 | 16373 | 10.08 | 16.7 | 3 |
| 135 | RN216_HUMAN | E3 ubiquitin-protein ligase RNF216 | 25 | 99342 | 4.83 | 1 | 1 |
| 136 | H2A1A_HUMAN | Histone H2A type 1-A | 25 | 14225 | 10.86 | 12.2 | 2 |
| 137 | MPP5_HUMAN | MAGUK p55 subfamily member 5 | 25 | 77246 | 5.77 | 1.5 | 1 |
| 138 | SRSF7_HUMAN | Serine/arginine-rich splicing factor 7 | 25 | 27350 | 11.83 | 14.7 | 4 |
| 139 | ZN473_HUMAN | Zinc finger protein 473 | 25 | 100118 | 8.63 | 1.1 | 1 |
| 140 | FILA_HUMAN | Filaggrin | 24 | 434922 | 9.24 | 0.9 | 3 |
| 141 | KDIS_HUMAN | Kinase D-interacting substrate of 220 kDa | 24 | 196419 | 6.18 | 1.5 | 2 |
| 142 | NIF3L_HUMAN | NIF3-like protein 1 | 24 | 41942 | 6.19 | 2.9 | 1 |
| 143 | O10J1_HUMAN | Olfactory receptor 10J1 | 24 | 35901 | 9.29 | 4.1 | 1 |
| 144 | RSF1_HUMAN | Remodeling and spacing factor 1 | 24 | 163720 | 4.94 | 0.8 | 1 |
| 145 | GRP75_HUMAN | Stress-70 protein, mitochondrial | 24 | 73635 | 5.87 | 5.3 | 4 |
| 146 | ZN878_HUMAN | Zinc finger protein 878 | 24 | 61500 | 9.55 | 3.6 | 2 |
| 147 | PDIA1_HUMAN | Protein disulfide-isomerase | 23 | 57081 | 4.76 | 7.5 | 3 |
| 148 | FA53C_HUMAN | Protein FAM53C | 23 | 43064 | 9 | 4.8 | 2 |
| 149 | AKA12_HUMAN | A-kinase anchor protein 12 | 22 | 191367 | 4.37 | 1 | 1 |
| 150 | IF5A1_HUMAN | Eukaryotic translation initiation factor 5A-1 | 22 | 16821 | 5.08 | 19.5 | 2 |
| 151 | GSDMA_HUMAN | Gasdermin-A | 22 | 49334 | 5.19 | 6.1 | 3 |
| 152 | ROA3_HUMAN | Heterogeneous nuclear ribonucleoprotein A3 | 22 | 39571 | 9.1 | 2.6 | 1 |
| 153 | PDE8B_HUMAN | High affinity cAMP-specific and IBMX-insensitive 3~,5~-cyclic phosphodiesterase 8B | 22 | 98916 | 6.35 | 1.7 | 2 |
| 154 | PTMA_HUMAN | Prothymosin alpha | 22 | 12196 | 3.69 | 9 | 1 |
| 155 | VP13C_HUMAN | Vacuolar protein sorting-associated protein 13C | 22 | 422124 | 6.38 | 0.8 | 4 |

**Supplemental Table 3 Chaperone Lon-associated proteins identified by in-solution-digestion and mass spectrometry**

|  | **Accession Number (Uniprot ID)** | **Protein name** | **Mascot Socre** | **Molecular Mass (Daltons)** | **pI** | **Sequence Coverage (%)** | **Matching Peptides** |
| --- | --- | --- | --- | --- | --- | --- | --- |
| 1 | LONM_HUMAN | Lon protease homolog, mitochondrial | 843 | 106422 | 6.01 | 24.5 | 18 |
| 2 | ENOA_HUMAN | Alpha-enolase | 574 | 47139 | 7.01 | 38 | 12 |
| 3 | TBA1A_HUMAN | Tubulin alpha-1A chain | 416 | 50104 | 4.94 | 35.9 | 11 |
| 4 | ACTB_HUMAN | Actin, cytoplasmic 1 | 380 | 41710 | 5.29 | 36.8 | 10 |
| 5 | RO52_HUMAN | E3 ubiquitin-protein ligase TRIM21 | 349 | 54135 | 5.98 | 27.8 | 11 |
| 6 | TBB5_HUMAN | Tubulin beta chain | 298 | 49639 | 4.78 | 24.5 | 8 |
| 7 | HNRPK_HUMAN | Heterogeneous nuclear ribonucleoprotein K | 282 | 50944 | 5.39 | 15.3 | 5 |
| 8 | HSP7C_HUMAN | Heat shock cognate 71 kDa protein | 272 | 70854 | 5.37 | 17.6 | 9 |
| 9 | ENOB_HUMAN | Beta-enolase | 269 | 46902 | 7.59 | 14.1 | 4 |
| 10 | HSP71_HUMAN | Heat shock 70 kDa protein 1A/1B | 257 | 70009 | 5.48 | 20 | 8 |
| 11 | CH60_HUMAN | 60 kDa heat shock protein, mitochondrial | 229 | 61016 | 5.7 | 12 | 5 |
| 12 | ENOG_HUMAN | Gamma-enolase | 210 | 47239 | 4.91 | 13.6 | 4 |
| 13 | TFG_HUMAN | Protein TFG | 200 | 43421 | 4.94 | 10.8 | 2 |
| 14 | NUCL_HUMAN | Nucleolin | 187 | 76568 | 4.6 | 13.5 | 8 |
| 15 | KCRB_HUMAN | Creatine kinase B-type | 163 | 42617 | 5.34 | 10 | 3 |
| 16 | H2B1B_HUMAN | Histone H2B type 1-B | 157 | 13942 | 10.31 | 31.7 | 4 |
| 17 | HSP76_HUMAN | Heat shock 70 kDa protein 6 | 150 | 70984 | 5.81 | 7.8 | 4 |
| 18 | ROA1_HUMAN | Heterogeneous nuclear ribonucleoprotein A1 | 143 | 38723 | 9.17 | 19.1 | 6 |
| 19 | NPM_HUMAN | Nucleophosmin | 142 | 32555 | 4.64 | 16.7 | 4 |
| 20 | PPIA_HUMAN | Peptidyl-prolyl cis-trans isomerase A | 131 | 18001 | 7.68 | 26.1 | 4 |
| 21 | TBA8_HUMAN | Tubulin alpha-8 chain | 131 | 50062 | 4.94 | 16.7 | 5 |
| 22 | EF1A1_HUMAN | Elongation factor 1-alpha 1 | 124 | 50109 | 9.1 | 16.5 | 6 |
| 23 | 1433Z_HUMAN | 14-3-3 protein zeta/delta | 118 | 27728 | 4.73 | 16.7 | 4 |
| 24 | THIO_HUMAN | Thioredoxin | 112 | 11730 | 4.82 | 21 | 2 |
| 25 | H2A1B_HUMAN | Histone H2A type 1-B/E | 108 | 14127 | 11.05 | 26.9 | 3 |
| 26 | ANXA2_HUMAN | Annexin A2 | 104 | 38580 | 7.57 | 4.7 | 1 |
| 27 | G3P_HUMAN | Glyceraldehyde-3-phosphate dehydrogenase | 104 | 36030 | 8.57 | 17 | 5 |
| 28 | GRP78_HUMAN | 78 kDa glucose-regulated protein | 100 | 72288 | 5.07 | 5.8 | 3 |
| 29 | RL6_HUMAN | 60S ribosomal protein L6 | 98 | 32708 | 10.59 | 8 | 2 |
| 30 | 1433E_HUMAN | 14-3-3 protein epsilon | 89 | 29155 | 4.63 | 11.4 | 3 |
| 31 | ACTBL_HUMAN | Beta-actin-like protein 2 | 87 | 41976 | 5.39 | 9 | 2 |
| 32 | HNRL1_HUMAN | Heterogeneous nuclear ribonucleoprotein U-like protein 1 | 87 | 95679 | 6.49 | 8.1 | 5 |
| 33 | RLA2_HUMAN | 60S acidic ribosomal protein P2 | 84 | 11658 | 4.42 | 16.5 | 1 |
| 34 | 1433G_HUMAN | 14-3-3 protein gamma | 80 | 28285 | 4.8 | 9.7 | 3 |
| 35 | PDIA6_HUMAN | Protein disulfide-isomerase A6 | 76 | 48091 | 4.95 | 3.2 | 1 |
| 36 | ATPB_HUMAN | ATP synthase subunit beta, mitochondrial | 75 | 56525 | 5.26 | 7.2 | 3 |
| 37 | RS24_HUMAN | 40S ribosomal protein S24 | 73 | 15413 | 10.79 | 9 | 1 |
| 38 | ROA2_HUMAN | Heterogeneous nuclear ribonucleoproteins A2/B1 | 70 | 37407 | 8.97 | 6.8 | 3 |
| 39 | EF1A2_HUMAN | Elongation factor 1-alpha 2 | 68 | 50438 | 9.11 | 8.4 | 4 |
| 40 | KPYM_HUMAN | Pyruvate kinase isozymes M1/M2 | 68 | 57900 | 7.96 | 7.2 | 3 |
| 41 | PRDX1_HUMAN | Peroxiredoxin-1 | 67 | 22096 | 8.27 | 20.1 | 4 |
| 42 | ENPL_HUMAN | Endoplasmin | 64 | 92411 | 4.76 | 2.7 | 2 |
| 43 | RL23A_HUMAN | 60S ribosomal protein L23a | 63 | 17684 | 10.44 | 8.3 | 1 |
| 44 | RS20_HUMAN | 40S ribosomal protein S20 | 62 | 13364 | 9.95 | 16 | 2 |
| 45 | RS2_HUMAN | 40S ribosomal protein S2 | 61 | 31305 | 10.25 | 7.8 | 2 |
| 46 | SMD3_HUMAN | Small nuclear ribonucleoprotein Sm D3 | 60 | 13907 | 10.33 | 15.1 | 2 |
| 47 | COF2_HUMAN | Cofilin-2 | 57 | 18725 | 7.66 | 11.4 | 2 |
| 48 | FLNA_HUMAN | Filamin-A | 55 | 280564 | 5.7 | 0.4 | 1 |
| 49 | PRDX2_HUMAN | Peroxiredoxin-2 | 54 | 21878 | 5.66 | 14.6 | 2 |
| 50 | CH10_HUMAN | 10 kDa heat shock protein, mitochondrial | 51 | 10925 | 8.89 | 13.7 | 1 |
| 51 | RL27A_HUMAN | 60S ribosomal protein L27a | 51 | 16551 | 11 | 7.4 | 1 |
| 52 | TCPE_HUMAN | T-complex protein 1 subunit epsilon | 51 | 59633 | 5.45 | 3 | 1 |
| 53 | STMN1_HUMAN | Stathmin | 49 | 17292 | 5.76 | 14.1 | 2 |
| 54 | RL12_HUMAN | 60S ribosomal protein L12 | 47 | 17808 | 9.48 | 5.5 | 1 |
| 55 | H13_HUMAN | Histone H1.3 | 46 | 22336 | 11.02 | 9 | 3 |
| 56 | RS4X_HUMAN | 40S ribosomal protein S4, X isoform | 46 | 29579 | 10.16 | 3.8 | 1 |
| 57 | TAGL2_HUMAN | Transgelin-2 | 45 | 22377 | 8.41 | 10.6 | 2 |
| 58 | RS25_HUMAN | 40S ribosomal protein S25 | 44 | 13734 | 10.12 | 7.2 | 1 |
| 59 | RBCC1_HUMAN | RB1-inducible coiled-coil protein 1 | 43 | 182975 | 5.3 | 0.5 | 1 |
| 60 | PLST_HUMAN | Plastin-3 | 41 | 70766 | 5.41 | 1.9 | 1 |
| 61 | ROAA_HUMAN | Heterogeneous nuclear ribonucleoprotein A/B | 41 | 36202 | 8.22 | 5.4 | 2 |
| 62 | DDX4_HUMAN | Probable ATP-dependent RNA helicase DDX4 | 40 | 79258 | 5.62 | 0.8 | 1 |
| 63 | HNRDL_HUMAN | Heterogeneous nuclear ribonucleoprotein D-like | 40 | 46409 | 9.59 | 4.3 | 2 |
| 64 | HNRPD_HUMAN | Heterogeneous nuclear ribonucleoprotein D0 | 40 | 38410 | 7.62 | 5.1 | 2 |
| 65 | HS90A_HUMAN | Heat shock protein HSP 90-alpha | 40 | 84607 | 4.94 | 6.8 | 5 |
| 66 | HS90B_HUMAN | Heat shock protein HSP 90-beta | 40 | 83212 | 4.97 | 3.7 | 3 |
| 67 | COF1_HUMAN | Cofilin-1 | 36 | 18491 | 8.22 | 10.2 | 2 |
| 68 | DLDH_HUMAN | Dihydrolipoyl dehydrogenase, mitochondrial | 36 | 54143 | 7.95 | 4.5 | 2 |
| 69 | NDUFS8_HUMAN | NADH dehydrogenase [ubiquinone] iron-sulfur protein 8, mitochondrial | 36 | 23690 | 6 | 5.7 | 2 |
| 70 | GOGB1_HUMAN | Golgin subfamily B member 1 | 36 | 375790 | 4.96 | 0.3 | 1 |
| 71 | RL35_HUMAN | 60S ribosomal protein L35 | 36 | 14543 | 11.04 | 15.4 | 2 |
| 72 | SYIM_HUMAN | Isoleucyl-tRNA synthetase, mitochondrial | 36 | 113719 | 6.78 | 1 | 1 |
| 73 | PCD18_HUMAN | Protocadherin-18 | 34 | 126071 | 4.99 | 1 | 1 |
| 74 | RLA1_HUMAN | 60S acidic ribosomal protein P1 | 34 | 11507 | 4.26 | 14 | 1 |
| 75 | SNCAP_HUMAN | Synphilin-1 | 34 | 100347 | 5.95 | 1.3 | 1 |
| 76 | ARI5A_HUMAN | AT-rich interactive domain-containing protein 5A | 32 | 64034 | 9.34 | 1.9 | 2 |
| 77 | CAPR1_HUMAN | Caprin-1 | 32 | 78318 | 5.14 | 1.7 | 1 |
| 78 | NDKA_HUMAN | Nucleoside diphosphate kinase A | 32 | 17138 | 5.83 | 11.2 | 1 |
| 79 | TPIS_HUMAN | Triosephosphate isomerase | 32 | 30772 | 5.65 | 10.1 | 1 |
| 80 | EF2_HUMAN | Elongation factor 2 | 31 | 95277 | 6.41 | 1.4 | 1 |
| 81 | K0494_HUMAN | EF-hand domain-containing protein KIAA0494 | 31 | 54997 | 5.98 | 2 | 1 |
| 82 | LEG12_HUMAN | Galectin-12 | 31 | 37518 | 9.3 | 3.6 | 1 |
| 83 | ROA3_HUMAN | Heterogeneous nuclear ribonucleoprotein A3 | 31 | 39571 | 9.1 | 4.8 | 2 |
| 84 | SRSF3_HUMAN | Serine/arginine-rich splicing factor 3 | 31 | 19318 | 11.64 | 8.5 | 1 |
| 85 | CENPE_HUMAN | Centromere-associated protein E | 30 | 316219 | 5.51 | 1.4 | 3 |
| 86 | SMG5_HUMAN | Protein SMG5 | 30 | 113855 | 5.63 | 0.6 | 1 |

**Supplemental Table 4 Chaperone Lon-associated proteins identified by in-solution-digestion and mass spectrometry**

|  | **Accession Number (Uniprot ID)** | **Protein name** | **Mascot Socre** | **Molecular Mass (Daltons)** | **pI** | **Sequence Coverage (%)** | **Matching Peptides** |
| --- | --- | --- | --- | --- | --- | --- | --- |
| 1 | MYH9_HUMAN | Myosin-9 | 1473 | 226392 | 5.5 | 31.1 | 71 |
| 2 | LONM_HUMAN | Lon protease homolog, mitochondrial | 931 | 106422 | 6.01 | 27.5 | 18 |
| 3 | MYH10_HUMAN | Myosin-10 | 624 | 228858 | 5.44 | 18.4 | 39 |
| 4 | RO52_HUMAN | E3 ubiquitin-protein ligase TRIM21 | 414 | 54135 | 5.98 | 33.1 | 12 |
| 5 | VIME_HUMAN | Vimentin | 344 | 53619 | 5.06 | 34.3 | 16 |
| 6 | CBX3_HUMAN | Chromobox protein homolog 3 | 326 | 20798 | 5.23 | 30.1 | 6 |
| 7 | CBX5_HUMAN | Chromobox protein homolog 5 | 290 | 22211 | 5.71 | 34.6 | 5 |
| 8 | ROA1_HUMAN | Heterogeneous nuclear ribonucleoprotein A1 | 285 | 38723 | 9.17 | 28.2 | 9 |
| 9 | TPM1_HUMAN | Tropomyosin alpha-1 chain | 261 | 32689 | 4.69 | 39.4 | 19 |
| 10 | MYH11_HUMAN | Myosin-11 | 210 | 227199 | 5.42 | 7.1 | 14 |
| 11 | TPM3_HUMAN | Tropomyosin alpha-3 chain | 207 | 32799 | 4.68 | 39.8 | 19 |
| 12 | TPM4_HUMAN | Tropomyosin alpha-4 chain | 206 | 28504 | 4.67 | 44.8 | 17 |
| 13 | HNRPC_HUMAN | Heterogeneous nuclear ribonucleoproteins C1/C2 | 203 | 33650 | 4.95 | 15.7 | 5 |
| 14 | CALM_HUMAN | Calmodulin | 197 | 16827 | 4.09 | 49 | 10 |
| 15 | TPM2_HUMAN | Tropomyosin beta chain | 194 | 32831 | 4.66 | 30.3 | 13 |
| 16 | ROA2_HUMAN | Heterogeneous nuclear ribonucleoproteins A2/B1 | 186 | 37407 | 8.97 | 20.7 | 6 |
| 17 | NPM_HUMAN | Nucleophosmin | 153 | 32555 | 4.64 | 21.8 | 5 |
| 18 | H2B1C_HUMAN | Histone H2B type 1-C/E/F/G/I | 149 | 13898 | 10.31 | 41.3 | 6 |
| 19 | H2B1B_HUMAN | Histone H2B type 1-B | 138 | 13942 | 10.31 | 40.5 | 5 |
| 20 | ACTB_HUMAN | Actin, cytoplasmic 1 | 135 | 41710 | 5.29 | 30.1 | 13 |
| 21 | HNRPK_HUMAN | Heterogeneous nuclear ribonucleoprotein K | 130 | 50944 | 5.39 | 9.9 | 3 |
| 22 | H12_HUMAN | Histone H1.2 | 110 | 21352 | 10.94 | 19.7 | 4 |
| 23 | H13_HUMAN | Histone H1.3 | 110 | 22336 | 11.02 | 18.6 | 17 |
| 24 | NUCL_HUMAN | Nucleolin | 104 | 76568 | 4.6 | 6.1 | 4 |
| 25 | SAFB1_HUMAN | Scaffold attachment factor B1 | 103 | 102580 | 5.32 | 2.3 | 1 |
| 26 | MYH14_HUMAN | Myosin-14 | 94 | 227732 | 5.52 | 3.7 | 7 |
| 27 | YBOX1_HUMAN | Nuclease-sensitive element-binding protein 1 | 92 | 35903 | 9.87 | 12.3 | 2 |
| 28 | HNRH1_HUMAN | Heterogeneous nuclear ribonucleoprotein H | 83 | 49198 | 5.89 | 10.9 | 3 |
| 29 | H4_HUMAN | Histone H4 | 83 | 11360 | 11.36 | 47.6 | 6 |
| 30 | GFAP_HUMAN | Glial fibrillary acidic protein | 79 | 49850 | 5.42 | 6.5 | 3 |
| 31 | MMAD_HUMAN | Methylmalonic aciduria and homocystinuria type D protein, mitochondrial | 77 | 32919 | 5.16 | 8.8 | 3 |
| 32 | GOGA1_HUMAN | Golgin subfamily A member 1 | 74 | 88130 | 5.24 | 15.3 | 8 |
| 33 | HNRPF_HUMAN | Heterogeneous nuclear ribonucleoprotein F | 74 | 45643 | 5.38 | 8.2 | 2 |
| 34 | RLA2_HUMAN | 60S acidic ribosomal protein P2 | 67 | 11658 | 4.42 | 11.3 | 1 |
| 35 | MYL3_HUMAN | Myosin light chain 3 | 65 | 21918 | 5.03 | 10.3 | 4 |
| 36 | TFG_HUMAN | Protein TFG | 63 | 43421 | 4.94 | 4.8 | 1 |
| 37 | DSG1_HUMAN | Desmoglein-1 | 57 | 113676 | 4.9 | 9.8 | 6 |
| 38 | HNRL1_HUMAN | Heterogeneous nuclear ribonucleoprotein U-like protein 1 | 53 | 95679 | 6.49 | 1.9 | 1 |
| 39 | BAF_HUMAN | Barrier-to-autointegration factor | 52 | 10052 | 5.81 | 13.5 | 1 |
| 40 | SRSF1_HUMAN | Serine/arginine-rich splicing factor 1 | 52 | 27728 | 10.37 | 4 | 1 |
| 41 | SAFB2_HUMAN | Scaffold attachment factor B2 | 51 | 107408 | 5.84 | 4.9 | 3 |
| 42 | SRSF7_HUMAN | Serine/arginine-rich splicing factor 7 | 51 | 27350 | 11.83 | 9.7 | 4 |
| 43 | ROA3_HUMAN | Heterogeneous nuclear ribonucleoprotein A3 | 49 | 39571 | 9.1 | 8.5 | 3 |
| 44 | HNRPD_HUMAN | Heterogeneous nuclear ribonucleoprotein D0 | 49 | 38410 | 7.62 | 2.8 | 1 |
| 45 | HNRDL_HUMAN | Heterogeneous nuclear ribonucleoprotein D-like | 49 | 46409 | 9.59 | 2.4 | 1 |
| 46 | ACTBL_HUMAN | Beta-actin-like protein 2 | 48 | 41976 | 5.39 | 4.8 | 1 |
| 47 | SKP1_HUMAN | S-phase kinase-associated protein 1 | 47 | 18646 | 4.4 | 7.4 | 1 |
| 48 | H2A1A_HUMAN | Histone H2A type 1-A | 46 | 14225 | 10.86 | 6.9 | 1 |
| 49 | IKIP_HUMAN | Inhibitor of nuclear factor kappa-B kinase-interacting protein | 46 | 39285 | 9.21 | 5.4 | 2 |
| 50 | RL34_HUMAN | 60S ribosomal protein L34 | 44 | 13284 | 11.48 | 6.8 | 2 |
| 51 | DESM_HUMAN | Desmin | 43 | 53503 | 5.21 | 5.5 | 3 |
| 52 | H11_HUMAN | Histone H1.1 | 42 | 21829 | 10.99 | 10.7 | 3 |
| 53 | RL29_HUMAN | 60S ribosomal protein L29 | 40 | 17741 | 11.66 | 9.4 | 1 |
| 54 | F184B_HUMAN | Protein FAM184B | 40 | 120969 | 5.87 | 3.7 | 5 |
| 55 | PCD18_HUMAN | Protocadherin-18 | 38 | 126071 | 4.99 | 2.8 | 2 |
| 56 | RLA1_HUMAN | 60S acidic ribosomal protein P1 | 37 | 11507 | 4.26 | 14 | 1 |
| 57 | CBX1_HUMAN | Chromobox protein homolog 1 | 36 | 21405 | 4.85 | 5.9 | 2 |
| 58 | NFM_HUMAN | Neurofilament medium polypeptide | 35 | 102411 | 4.9 | 1.9 | 2 |
| 59 | EWS_HUMAN | RNA-binding protein EWS | 34 | 68436 | 9.37 | 1.4 | 1 |
| 60 | CENPE_HUMAN | Centromere-associated protein E | 33 | 316219 | 5.51 | 1.2 | 3 |
| 61 | KCAB1_HUMAN | Voltage-gated potassium channel subunit beta-1 | 33 | 46534 | 9.1 | 1.4 | 1 |
| 62 | DESP_HUMAN | Desmoplakin | 31 | 331569 | 6.44 | 2.3 | 8 |
| 63 | RBBP4_HUMAN | Histone-binding protein RBBP4 | 31 | 47626 | 4.74 | 6.6 | 2 |
| 64 | CNTP4_HUMAN | Contactin-associated protein-like 4 | 30 | 145182 | 6.22 | 3.4 | 7 |
| 65 | DDX46_HUMAN | Probable ATP-dependent RNA helicase DDX46 | 30 | 117290 | 9.33 | 5.5 | 4 |
| 66 | MUTYH_HUMAN | A/G-specific adenine DNA glycosylase | 29 | 60031 | 8.99 | 2.4 | 1 |
| 67 | NFH_HUMAN | Neurofilament heavy polypeptide | 29 | 112411 | 5.99 | 4.2 | 2 |
| 68 | NFL_HUMAN | Neurofilament light polypeptide | 29 | 61479 | 4.64 | 3.1 | 2 |
| 69 | RL13_HUMAN | 60S ribosomal protein L13 | 28 | 24247 | 11.65 | 5.2 | 1 |
| 70 | RL23A_HUMAN | 60S ribosomal protein L23a | 28 | 17684 | 10.44 | 8.3 | 1 |
| 71 | DYH1_HUMAN | Dynein heavy chain 1, axonemal | 28 | 493637 | 5.65 | 1 | 3 |
| 72 | FYCO1_HUMAN | FYVE and coiled-coil domain-containing protein 1 | 28 | 166879 | 4.86 | 4.5 | 5 |
| 73 | PRC1_HUMAN | Protein regulator of cytokinesis 1 | 28 | 71562 | 6.29 | 1 | 1 |
| 74 | STRN4_HUMAN | Striatin-4 | 28 | 80546 | 5.21 | 7.4 | 3 |
| 75 | RL4_HUMAN | 60S ribosomal protein L4 | 27 | 47667 | 11.07 | 8 | 3 |
| 76 | APOA_HUMAN | Apolipoprotein(a) | 27 | 500995 | 5.58 | 0.2 | 1 |
| 77 | GAGD2_HUMAN | G antigen family D member 2 | 27 | 9072 | 9.65 | 7.4 | 1 |
| 78 | MPIP3_HUMAN | M-phase inducer phosphatase 3 | 27 | 53331 | 6.34 | 3 | 2 |
| 79 | LMBL2_HUMAN | Lethal(3)malignant brain tumor-like protein 2 | 26 | 79059 | 6.4 | 0.9 | 1 |
| 80 | SYNE2_HUMAN | Nesprin-2 | 26 | 795944 | 5.26 | 1.5 | 9 |
| 81 | WDR16_HUMAN | WD repeat-containing protein 16 | 26 | 68255 | 6.51 | 1 | 1 |
| 82 | CASPE_HUMAN | Caspase-14 | 25 | 27662 | 5.44 | 9.1 | 3 |
| 83 | TESK2_HUMAN | Dual specificity testis-specific protein kinase 2 | 25 | 63599 | 6.63 | 5.1 | 4 |
| 84 | PIGF_HUMAN | Phosphatidylinositol-glycan biosynthesis class F protein | 25 | 24873 | 8.88 | 3.7 | 1 |
| 85 | UBR3_HUMAN | E3 ubiquitin-protein ligase UBR3 | 24 | 212296 | 5.74 | 1.1 | 5 |
| 86 | UTY_HUMAN | Histone demethylase UTY | 24 | 149453 | 7.91 | 3 | 2 |
| 87 | H31T_HUMAN | Histone H3.1t | 24 | 15499 | 11.13 | 5.1 | 1 |
| 88 | KDM6A_HUMAN | Lysine-specific demethylase 6A | 24 | 154079 | 7.17 | 2.1 | 2 |
| 89 | S12A1_HUMAN | Solute carrier family 12 member 1 | 24 | 121372 | 7.18 | 0.5 | 1 |
| 90 | WDR90_HUMAN | WD repeat-containing protein 90 | 24 | 187317 | 6.56 | 6.5 | 5 |
| 91 | ZN619_HUMAN | Zinc finger protein 619 | 24 | 63281 | 8.72 | 5.2 | 2 |
| 92 | KIF14_HUMAN | Kinesin-like protein KIF14 | 23 | 186375 | 8.06 | 3.9 | 4 |
| 93 | NIF3L_HUMAN | NIF3-like protein 1 | 23 | 41942 | 6.19 | 2.9 | 1 |
| 94 | XPO2_HUMAN | Exportin-2 | 22 | 110346 | 5.51 | 0.8 | 1 |
| 95 | COQ6_HUMAN | Ubiquinone biosynthesis monooxygenase COQ6 | 22 | 50838 | 6.81 | 1.9 | 1 |

**Supplemental Table 5 Chaperone Lon-associated proteins identified by in-solution-digestion and mass spectrometry**

|  | **Accession Number (Uniprot ID)** | **Protein name** | **MW (Da)** |
| --- | --- | --- | --- |
| 1 | DESP_HUMAN | Desmoplakin | 331569 |
| 2 | VIME_HUMAN | Vimentin | 53619 |
| 3 | MYH9_HUMAN | Myosin-9 | 226392 |
| 4 | DSG1_HUMAN | Desmoglein-1 | 113676 |
| 5 | HSP71_HUMAN | Heat shock 70 kDa protein 1A/1B | 70009 |
| 6 | GRP75_HUMAN | Stress-70 protein, mitochondrial | 73635 |
| 7 | DLDH_HUMAN | Dihydrolipoyl dehydrogenase, mitochondrial | 54143 |
| 8 | TPM1_HUMAN | Tropomyosin alpha-1 chain | 32689 |
| 9 | HNRPC_HUMAN | Heterogeneous nuclear ribonucleoproteins C1/C2 | 33650 |
| 10 | RLA2_HUMAN | 60S acidic ribosomal protein P2 | 11658 |
| 11 | TPM3_HUMAN | Tropomyosin alpha-3 chain | 32799 |
| 12 | CH60_HUMAN | 60 kDa heat shock protein, mitochondrial | 61016 |
| 13 | H2B1B_HUMAN | Histone H2B type 1-B | 13942 |
| 14 | LONM_HUMAN | Lon protease homolog, mitochondrial | 106422 |
| 15 | HSP76_HUMAN | Heat shock 70 kDa protein 6 | 70984 |
| 16 | ROA1_HUMAN | Heterogeneous nuclear ribonucleoprotein A1 | 38723 |
| 17 | TPIS_HUMAN | Triosephosphate isomerase | 30772 |
| 18 | HSP7C_HUMAN | Heat shock cognate 71 kDa protein | 70854 |
| 19 | H13_HUMAN | Histone H1.3 | 22336 |
| 20 | RO52_HUMAN | E3 ubiquitin-protein ligase TRIM21 | 54135 |
| 21 | CASPE_HUMAN | Caspase-14 | 27662 |
| 22 | TPM4_HUMAN | Tropomyosin alpha-4 chain | 28504 |
| 23 | NPM_HUMAN | Nucleophosmin | 32555 |
| 24 | HNRPK_HUMAN | Heterogeneous nuclear ribonucleoprotein K | 50944 |
| 25 | GRP78_HUMAN | 78 kDa glucose-regulated protein | 72288 |
| 26 | CBX3_HUMAN | Chromobox protein homolog 3 | 20798 |
| 27 | YBOX1_HUMAN | Nuclease-sensitive element-binding protein 1 | 35903 |
| 28 | GOGA1_HUMAN | Golgin subfamily A member 1 | 88130 |
| 29 | HNRH1_HUMAN | Heterogeneous nuclear ribonucleoprotein H | 49198 |
| 30 | NFH_HUMAN | Neurofilament heavy polypeptide | 112411 |
| 31 | MMAD_HUMAN | Methylmalonic aciduria and homocystinuria type D protein, mitochondrial | 32919 |
| 32 | ACTBL_HUMAN | Beta-actin-like protein 2 | 41976 |
| 33 | ACTB_HUMAN | Actin, cytoplasmic 1 | 41710 |
| 34 | MYH10_HUMAN | Myosin-10 | 228858 |
| 35 | CALM_HUMAN | Calmodulin | 16827 |
| 36 | CBX1_HUMAN | Chromobox protein homolog 1 | 21405 |
| 37 | HNRPF_HUMAN | Heterogeneous nuclear ribonucleoprotein F | 45643 |
| 38 | ROA2_HUMAN | Heterogeneous nuclear ribonucleoproteins A2/B1 | 37407 |
| 39 | DESM_HUMAN | Desmin | 53503 |
| 40 | G3P_HUMAN | Glyceraldehyde-3-phosphate dehydrogenase | 36030 |
| 41 | TBA1A_HUMAN | Tubulin alpha-1A chain | 50104 |
| 42 | TFG_HUMAN | Protein TFG | 43421 |
| 43 | THIO_HUMAN | Thioredoxin | 11730 |
| 44 | H11_HUMAN | Histone H1.1 | 21829 |
| 45 | PRDX1_HUMAN | Peroxiredoxin-1 | 22096 |
| 46 | ENOA_HUMAN | Alpha-enolase | 47139 |
| 47 | RBBP4_HUMAN | Histone-binding protein RBBP4 | 47626 |
| 48 | RLA1_HUMAN | 60S acidic ribosomal protein P1 | 11507 |
| 49 | MYL3_HUMAN | Myosin light chain 3 | 21918 |
| 50 | HNRL1_HUMAN | Heterogeneous nuclear ribonucleoprotein U-like protein 1 | 95679 |
| 51 | RL23A_HUMAN | 60S ribosomal protein L23a | 17684 |
| 52 | COF1_HUMAN | Cofilin-1 | 18491 |
| 53 | RL29_HUMAN | 60S ribosomal protein L29 | 17741 |
| 54 | NFL_HUMAN | Neurofilament light polypeptide | 61479 |
| 55 | NFM_HUMAN | Neurofilament medium polypeptide | 102411 |
| 56 | HNRPD_HUMAN | Heterogeneous nuclear ribonucleoprotein D0 | 38410 |
| 57 | HNRDL_HUMAN | Heterogeneous nuclear ribonucleoprotein D-like | 46409 |
| 58 | H4_HUMAN | Histone H4 | 11360 |
| 59 | IKIP_HUMAN | Inhibitor of nuclear factor kappa-B kinase-interacting protein | 39285 |
| 60 | PCD18_HUMAN | Protocadherin-18 | 126071 |
| 61 | STRN4_HUMAN | Striatin-4 | 80546 |
| 62 | EF1A1_HUMAN | Elongation factor 1-alpha 1 | 50109 |
| 63 | CENPE_HUMAN | Centromere-associated protein E | 316219 |
| 64 | XPO2_HUMAN | Exportin-2 | 110346 |
| 65 | COQ6_HUMAN | Ubiquinone biosynthesis monooxygenase COQ6 | 50838 |
| 66 | 1433E_HUMAN | 14-3-3 protein epsilon | 29155 |
| 67 | 1433G_HUMAN | 14-3-3 protein gamma | 28285 |
| 68 | 1433Z_HUMAN | 14-3-3 protein zeta/delta | 27728 |
| 69 | H2A1A_HUMAN | Histone H2A type 1-A | 14225 |
| 70 | SRSF7_HUMAN | Serine/arginine-rich splicing factor 7 | 27350 |
| 71 | ANXA2_HUMAN | Annexin A2 | 38580 |
| 72 | ATPB_HUMAN | ATP synthase subunit beta, mitochondrial | 56525 |
| 73 | NIF3L_HUMAN | NIF3-like protein 1 | 41942 |
| 74 | NDUFS8_HUMAN | NADH dehydrogenase [ubiquinone] iron-sulfur protein 8, mitochondrial | 23690 |
| 75 | ROA3_HUMAN | Heterogeneous nuclear ribonucleoprotein A3 | 39571 |
| 76 | TPM2_HUMAN | Tropomyosin beta chain | 49639 |

**Supplemental Table 6 Chaperone Lon-associated proteins identified by in-gel-digestion and in-solution-digestion mass spectrometry**

|  | **Accession Number (Uniprot ID)** | **Protein name** | **Molecular Mass (Daltons)** |
| --- | --- | --- | --- |
| 1 | MYH9_HUMAN | Myosin-9 | 226392 |
| 2 | DESP_HUMAN | Desmoplakin | 331569 |
| 3 | LONM_HUMAN | Lon protease homolog, mitochondrial | 106422 |
| 4 | MYH10_HUMAN | Myosin-10 | 228858 |
| 5 | HSP71_HUMAN | Heat shock 70 kDa protein 1A/1B | 70009 |
| 6 | HSP7C_HUMAN | Heat shock cognate 71 kDa protein | 70854 |
| 7 | HSP76_HUMAN | Heat shock 70 kDa protein 6 | 70984 |
| 8 | HS71L_HUMAN | Heat shock 70 kDa protein 1-like | 70331 |
| 9 | GRP78_HUMAN | 78 kDa glucose-regulated protein | 72288 |
| 10 | TBA8_HUMAN | Tubulin alpha-8 chain | 50062 |
| 11 | NFH_HUMAN | Neurofilament heavy polypeptide | 112411 |
| 12 | ACTBL_HUMAN | Beta-actin-like protein 2 | 41976 |
| 13 | PCD18_HUMAN | Protocadherin-18 | 126071 |
| 14 | TBB2A_HUMAN | Tubulin beta-2A chain | 49875 |
| 15 | HNRPU_HUMAN | Heterogeneous nuclear ribonucleoprotein U | 90528 |
| 16 | GRP75_HUMAN | Stress-70 protein, mitochondrial | 73635 |
| 17 | HNRPC_HUMAN | Heterogeneous nuclear ribonucleoproteins C1/C2 | 33650 |
| 18 | HNRPK_HUMAN | Heterogeneous nuclear ribonucleoprotein K | 50944 |
| 19 | HNRH1_HUMAN | Heterogeneous nuclear ribonucleoprotein H | 49198 |
| 20 | NFL_HUMAN | Neurofilament light polypeptide | 61479 |
| 21 | NFM_HUMAN | Neurofilament medium polypeptide | 102411 |
