# Supplementary material for: Mitochondrial Lon regulates apoptosis through the association with Hsp60–mtHsp70 complex
Source: Cell Death Dis. 2015 Feb 12;6(2):e1642–. doi: 10.1038/cddis.2015.9 (PMC4669791; doi:10.1038/cddis.2015.9)

**Supporting Information**

**Mitochondrial Lon regulates apoptosis through the association with Hsp60-mtHsp70 complex**

Ting-Yu Kao^1^ , Yi-Chieh Chiu^1,2^, Wei-Cheng Fang^2,3,4^, Chieh-Wen Cheng^2^, Chan-Yen Kuo^2^, Hsueh-Fen Juan^5,6^, Shih-Hsiung Wu^3,4,7^*, and Alan Yueh-Luen Lee^2^*

*: Corresponding author:

**Legends for Supplemental Figures**

**Supplemental Figure 1. Characterization of 293 cells stably overexpressing mitochondrial Lon protease (293/Lon).**

**A. Lon-myc is successfully overexpressed in 293/Lon cells.**

The survived 293/Lon cells were collected to check the expression of human Lon protease by western blotting. The expression of Lon and myc-Lon were detected by Western blotting using an antibody to Lon and myc, respectively. An antibody to Actin was used as a loading control

**B. Mitochondrial localization of overexpressed myc-Lon in 293/Lon cells verified by immunofluorescence**

293 or 293/Lon cells were fixed and immunostained by anti-Lon antibody (green), MitoTracker (red), and anti-Myc antibody (red). Myc-Lon is specifically expressed in the mitochondria of 293/Lon cells. MitoTracker, a mitochondrion selective dye, was used as an indicator to demonstrate mitochondrial localization of Lon. DNA was stained with DAPI (blue).

**Supplemental Figure 2. Identification of Lon-associated proteins by in-solution digestion shotgun proteomics**

**A.** The structure of anti-myc agarose beads used in this study in which agarose covalently conjugates with anti-myc antibody.

**B. 6 M urea is not able to release anti-myc antibodies from the agarose beads.** The antibody elution test was performed by two different methods. The agarose beads were either boiled at 95°C to denature all proteins on the beads or washed by 6 M urea. The result shows that the antibodies will not be eluted from the agarose beads by washing of 6 M urea.

**Supplemental Figure 3. Venn diagram and bioinformatics analysis of Lon-interacting proteins identified by in-solution digestion shotgun proteomics**

**A.** Total amount of candidate proteins from three independent experiments (246 proteins) were obtained after exclusion of non-specific binding of anti-myc agarose beads.

**B.** Functional analyses were generated through Ingenuity Pathways Analysis (Ingenuity Systems, www.ingenuity.com). The first two enriched function categories are cancer and cell death. Threshold bar shows cut-off point of significance *P* < 0.05, −log (*P*-value) of 1.3 determined by the right-tailed Fisher’s exact test.

**C. The interaction network of Lon-associated proteins involved in mtHsp70-Hsp60 complex**. The network is generated by direct interaction algorithm of MetaCore (GeneGo) software using the list of hLon-associated proteins identified by shotgun proteomics analysis. Individual proteins are represented as *nodes* and the *edges* define the relationships of the nodes: the *arrowheads* indicate the direction of the interaction.

**Supplemental Figure 4. NDUFS8 interacts with Lon validated by co-immunoprecipitation and immunofluorescence experiment**

**A. Lon interacts with NDUFS8 shown by co-immunoprecipitation.**

293 cells were stably transfected with plasmid encoding Myc-Lon followed by co-immunoprecipitation with anti-Lon and anti-NDUFS8, respectively. The immunoprecipitation complex was analyzed by Western blotting using indicated antibodies.

**B. The interaction between mitochondrial Lon and NDUFS8 was verified by confocal immunofluorescence**

293/Lon cells were fixed and immunostained by anti-NDUFS8 (green) and anti-Lon (red) antibodies. DNA was stained with DAPI (blue).

**Supplemental Figure 5. Endogenous interaction between Lon and Hsp60-mtHsp70.** 293 cells were exposed to 200 μM H_2_O_2_ for 1 hour and direct stained by anti-Lon (green), anti-mtHsp70 (red), anti-Hsp60 (green), or anti-myc (red) as indicated, following image capture by fluorescence microscopy. DNA was stained with DAPI (blue).

**Supplemental Figure 6. Down-regulation of Lon in 293 cells by shRNA using retrovirus system**

Lon expression was inhibited by shRNA, sh-1 and sh-2, using retrovirus transfection system. The bar graph shown in the bottom represents Lon levels normalized against actin.


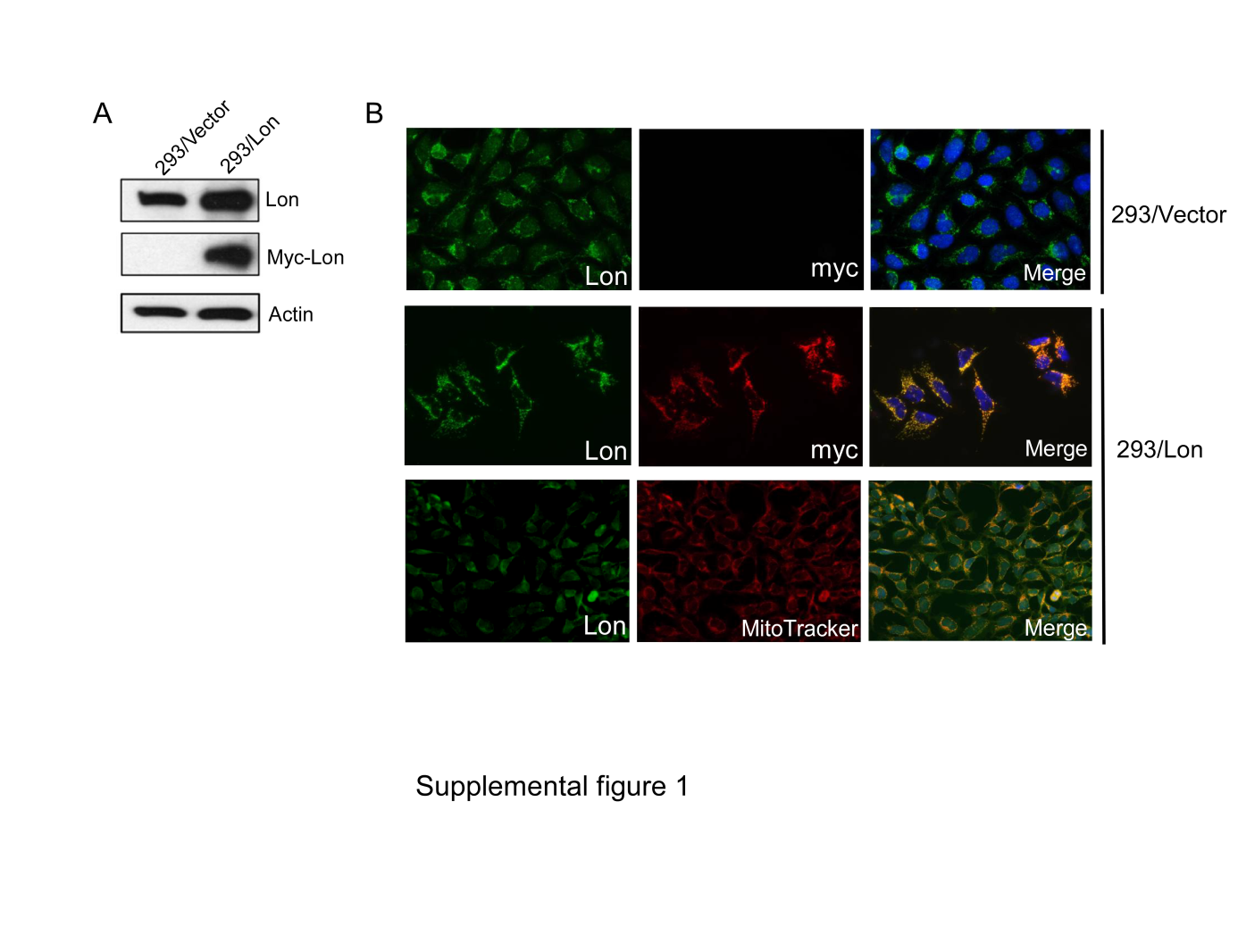


**
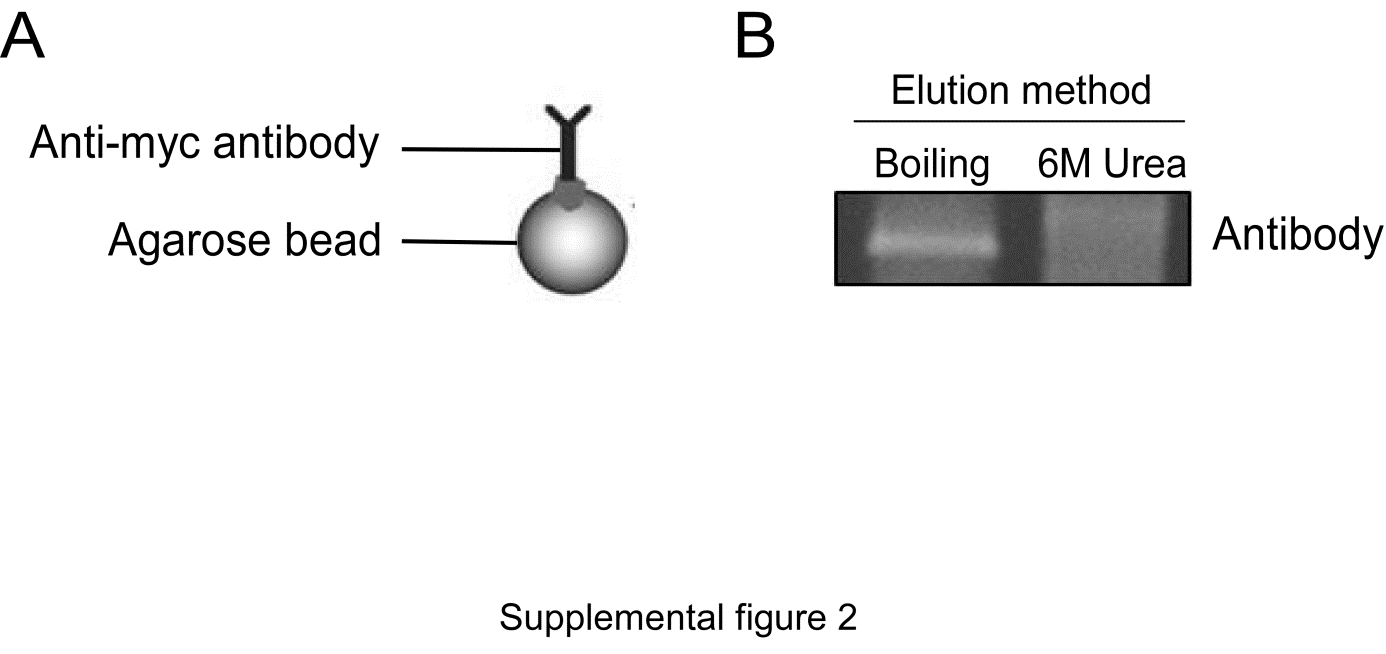
**

**
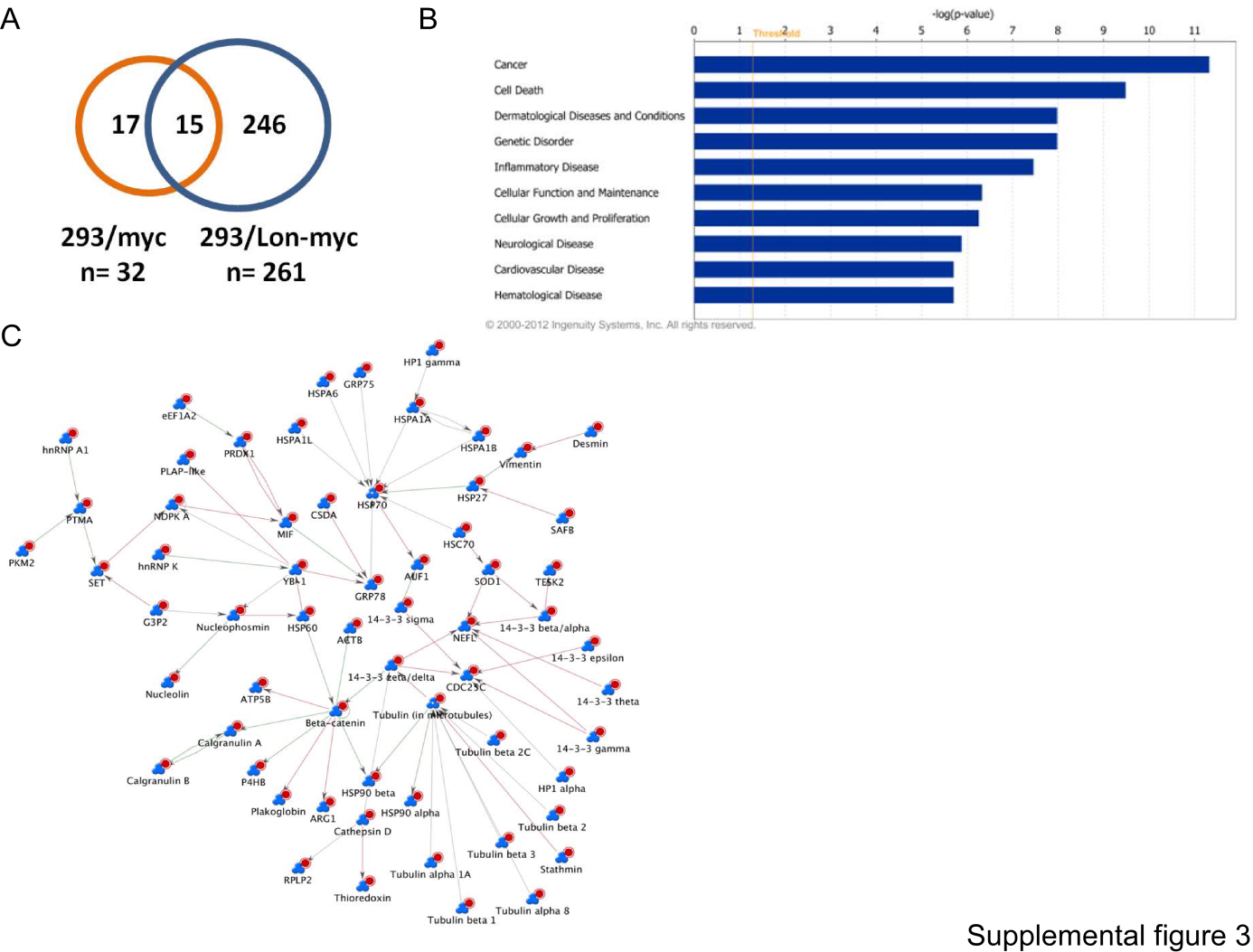
**


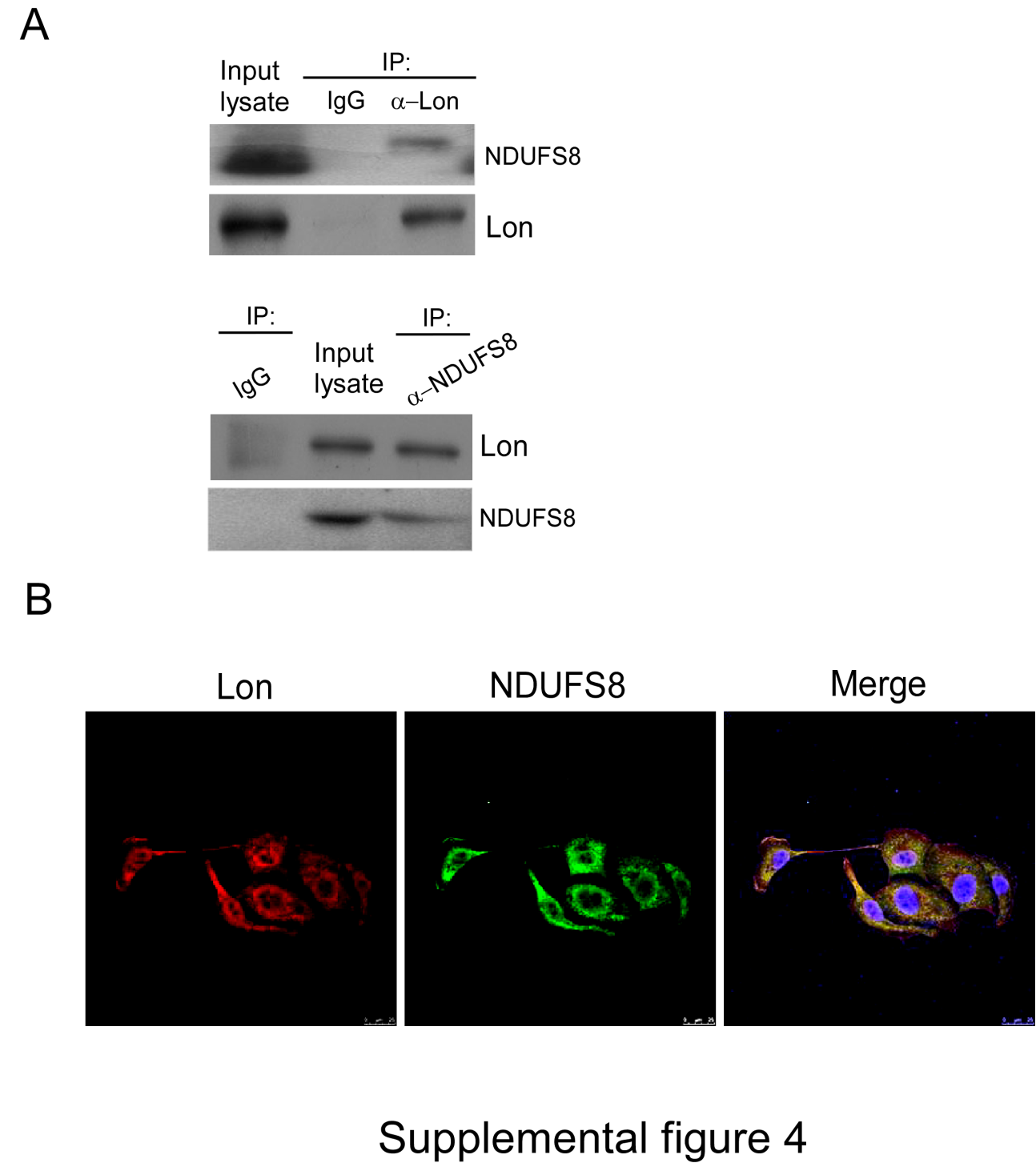


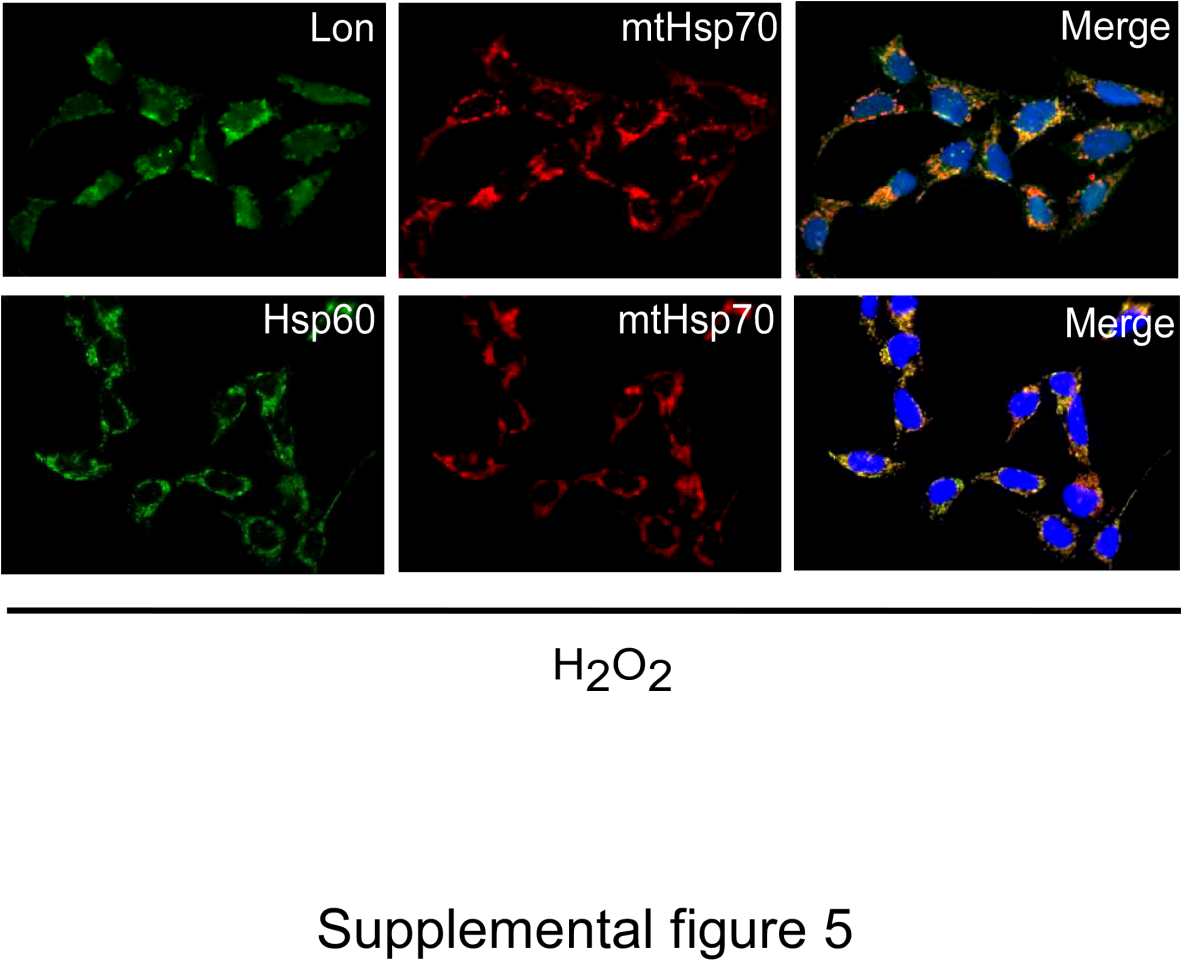


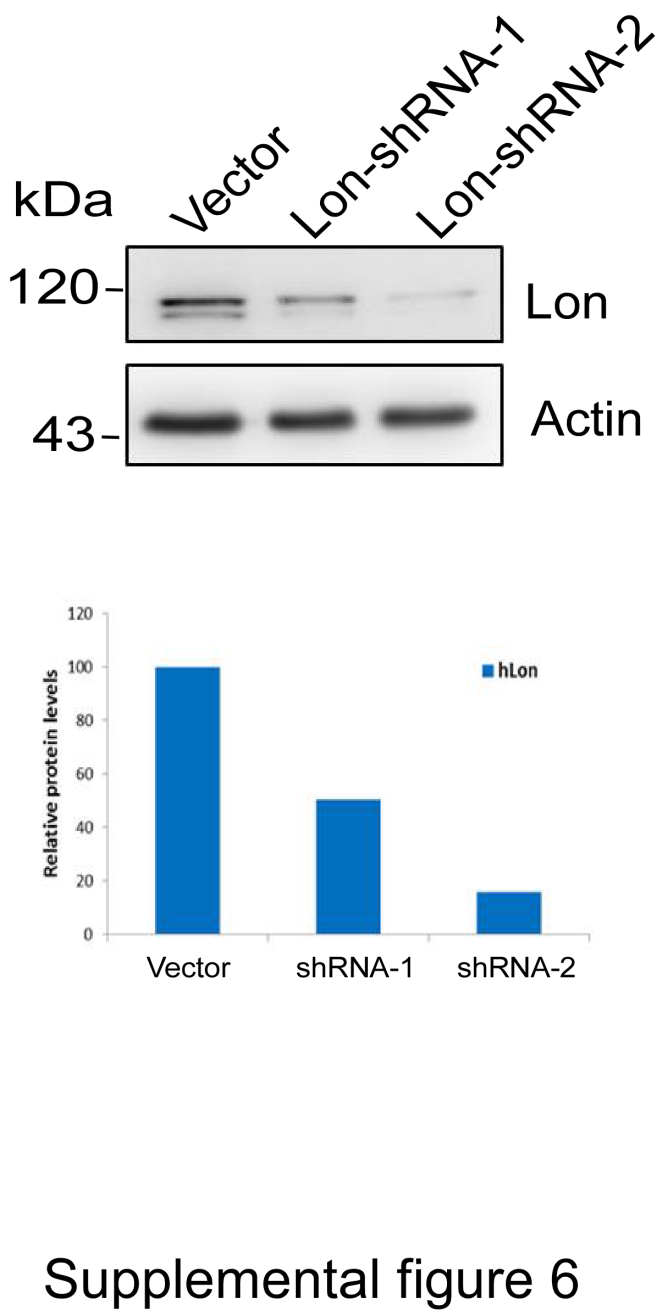

Supplement: Supplementary Information [file cddis20159x2.doc]
